# Advancing Research and Treatment: An Overview of Clinical Trials in Myalgic Encephalomyelitis/Chronic Fatigue Syndrome (ME/CFS) and Future Perspectives

**DOI:** 10.3390/jcm13020325

**Published:** 2024-01-06

**Authors:** Katharine A. Seton, José A. Espejo-Oltra, Karen Giménez-Orenga, Rik Haagmans, Donia J. Ramadan, Jesper Mehlsen

**Affiliations:** 1 Quadram Institute Bioscience, Norwich Research Park, Norwich NR4 7UQ, UK; rik.haagmans@quadram.ac.uk; 2 Max Delbrück Center for Molecular Medicine, Robert-Rössle-Straße 10, 13125 Berlin, Germany; joseandres.espejooltra@mdc-berlin.de; 3 Department of Pathology, School of Health Sciences, Universidad Católica de Valencia, San Vicente Mártir, 46001 Valencia, Spain; 4 Escuela de Doctorado, Universidad Católica de Valencia, San Vicente Mártir, 46001 Valencia, Spain; karen.gimenez@mail.ucv.es; 5 Norwich Medical School, University of East Anglia, Norwich NR4 7TJ, UK; 6 Department of Medical Genetics, Oslo University Hospital and University of Oslo, Kirkeveien 166, 0450 Oslo, Norway; doniajr@uio.no; 7 Surgical Pathophysiology Unit, Rigshospitalet, University of Copenhagen, 2100 Copenhagen, Denmark; jesper.mehlsen.01@regionh.dk

**Keywords:** myalgic encephalomyelitis/chronic fatigue syndrome, treatment, therapies, clinical trial, immunological, metabolic, gastrointestinal, neurological, neuroendocrine, outcome measure

## Abstract

Myalgic encephalomyelitis/chronic fatigue syndrome (ME/CFS) is a chronic, debilitating, and multi-faceted illness. Heterogenous onset and clinical presentation with additional comorbidities make it difficult to diagnose, characterize, and successfully treat. Current treatment guidelines focus on symptom management, but with no clear target or causative mechanism, remission rates are low, and fewer than 5% of patients return to their pre-morbid activity levels. Therefore, there is an urgent need to undertake robust clinical trials to identify effective treatments. This review synthesizes insights from clinical trials exploring pharmacological interventions and dietary supplements targeting immunological, metabolic, gastrointestinal, neurological, and neuroendocrine dysfunction in ME/CFS patients which require further exploration. Additionally, the trialling of alternative interventions in ME/CFS based on reported efficacy in the treatment of illnesses with overlapping symptomology is also discussed. Finally, we provide important considerations and make recommendations, focusing on outcome measures, to ensure the execution of future high-quality clinical trials to establish clinical efficacy of evidence-based interventions that are needed for adoption in clinical practice.

## 1. Introduction

Myalgic encephalomyelitis/chronic fatigue syndrome (ME/CFS) is a debilitating and heterogenous illness that affects between 17–24 million people worldwide [1]. Diagnosing ME/CFS remains challenging due to no available tests or biomarker [2]. A causative agent has not been identified, with patients reporting a range of events occurring prior to the onset of ME/CFS, including infectious illnesses, stress, or major life events and exposure to chemical or environmental toxins [3]. Variations in symptoms and severity, the presence of comorbid illnesses, and the large number of ME/CFS case definitions further contribute to the heterogeneity among ME/CFS patients [4,5,6]. Consequently, it has been challenging for researchers to identify consistent pathophysiological changes that occur across all ME/CFS patients with our understanding of disturbances in the immune, metabolic, gastrointestinal (GI), nervous, and endocrine systems remaining incomplete [7]. There is currently no cure or FDA-recommended treatment for ME/CFS. Consequently, fewer than 5% of patients return to their pre-morbid activity levels [3]. ME/CFS has a substantial economic burden due to healthcare costs and loss of productivity [8], with 75% of patients unable to work [9] and 25% confined to their house or bed [10].

In the past, cognitive behavioural therapy (CBT) and graded exercise therapy (GET) were recommended by health agencies for the treatment of ME/CFS [11]. Reanalysis of the 2011 PACE (pacing, graded activity, and cognitive behaviour therapy; a randomised evaluation) trial refuted original claims of the safety and efficacy of GET and CBT (reviewed in [12]). Consequently, treatment guidelines have undergone revisions, removing GET as a recommended therapy, while CBT is now provided as a supplementary approach to alleviate the distress related to chronic illness [13,14,15]. Health agencies recommend a multidisciplinary, individualised approach to treat or manage the symptoms patients experience, utilising both pharmacological and non-pharmacological treatments [13,14]. In addition, comorbid illnesses such as anxiety, depression, fibromyalgia (FM), irritable bowel syndrome (IBS), and migraine headaches are common in ME/CFS patients [3]. Treatment and management of comorbid illnesses need to be considered carefully, due to some recommended treatments for the comorbid illnesses having detrimental effects on ME/CFS symptoms [13,15].

This review evaluates past clinical trials (CTs) in ME/CFS, identifying gaps requiring further investigation and providing recommendations for planning and designing robust future CTs into ME/CFS.

## 2. Infection

Reports of outbreaks and sporadic cases of ME/CFS have suggested infections as potential triggers for the disease [16,17]. Herpesviruses, particularly Epstein–Barr virus (EBV), human herpesvirus-6 types A and B (HHV-6), human herpesvirus 7 (HHV-7), and human cytomegalovirus (HCMV) are the most likely viruses associated with ME/CFS [18,19,20] based on findings of increased viral load and virus-specific antibody titres [19,21,22]. Herpesvirus reactivation may contribute to ME/CFS pathophysiology via inflammation and immune dysregulation [23,24]. CTs have evaluated the effect of antiviral therapy and immunomodulators in ME/CFS patients (Table S1).

### 2.1. Nucleoside Analogues

Nucleoside analogues used for the treatment of herpesviruses consist of a “prodrug” that is selectively metabolized by viral thymidine kinase to its active form, which inhibits viral replication [25,26]. The first antiviral based on a nucleoside analogue tested in ME/CFS patients was acyclovir (ACV) [25,26,27], active against herpes simplex virus 1 (HSV-1) and 2 (HSV-2) but less so against EBV and HCMV [28]. A double-blind, cross-over, placebo-controlled study administrating intravenous ACV for one month to twenty-four ME/CFS patients revealed no significant improvement in symptoms compared to placebo [27]. Given the low solubility in water, low oral bioavailability, and short plasma half-life of ACV, more soluble forms were developed, like the L-valyl ester from ACV, termed valacyclovir (VACV) [25]. VACV has improved GI absorption and is three times more orally bioavailable than ACV [25,26]. Two pilot studies found administering VACV over six months improved physical activity in ME/CFS patients with elevated EBV antibodies [29,30]. However, patients with elevated HCMV antibodies did not improve [29]. 

Another antiviral structurally related to ACV is ganciclovir (GCV). Limited oral bioavailability of GCV prompted the development of its valine ester, valganciclovir (VGCV), with improved GI absorption [31]. The benefits of VGCV treatment were evidenced in (i) a 24-week open-label pilot study (OPT) performed in twelve ME/CFS patients with elevated antibody titres to both HHV-5 and EBV [32], (ii) a review of medical records of ME/CFS patients with positive EBV, HHV-6, and HCMV serum antibody titres administered VGCV for at least 6 months [33], (iii) a retrospective chart review performed on sixty-one ME/CFS patients with high HHV-6 and EBV antibody titres for 3 weeks [34], and (iv) a randomized double-blind, placebo-controlled trial (RCT) performed in thirty ME/CFS patients with high HHV-6 and EBV antibody titres for 6 months [35]. Of note, some studies reported a severe initial worsening of ME/CFS symptoms that subsequently improved [32,33,35]. Otherwise, no severe effects have been reported. A recent retrospective study comparing the efficacy in treating reactivated HHV-6 and HHV-7 infections with VACV, VGCV, and artesunate, an antiviral that targets both the herpesvirus and susceptible host cell, found that all antivirals were able to reduce HHV-6 and HHV-7 levels in ME/CFS patients, with artesunate being the most effective [36].

### 2.2. Immunomodulators

Immunomodulators enhance the immune response to viral infections. Rintatolimod, sold under the trade name Ampligen, consists of a double-stranded RNA, modified to exclusively bind toll-like receptor 3 (TLR3) [37] which acts as a first line of defence against microbial pathogens by recognizing bacteria or virus nucleic acids [38]. Rintatolimod binding to TLR3 activates dendritic cells, potentiating humoral and cell-mediated responses in a MyD88-independent TRIF pathway, preventing systemic cytokine induction, thereby reducing adverse effects [39]. In a Phase I CT, rintatolimod showed improvement in cognitive impairment [40]. In a Phase II RCT, rintatolimod was well tolerated with a significant association between greater functional impairment and HHV-6 positivity identified [41]. Finally, a Phase III RCT confirmed significant improvements in exercise tolerance in ME/CFS patients, particularly for patients with symptom durations between two and eight years [42].

### 2.3. Future Directions

The efficacy of VACV, VGVC, and artesunate should be confirmed in larger cohorts of ME/CFS patients with high virus antibody titres. Rintatolimod is the only drug to date that has undergone a Phase III clinical trial with confirmed improvements in ME/CFS patients. It has been approved for use in severe ME/CFS patients in Argentina [43], but it is still classified as an experimental drug and not approved for use in other countries (reviewed in [44]). Alternative antivirals should be tested in ME/CFS patients, such as luteolin, which targets EBV and has been proven effective in treating pain, anxiety, depression, fatigue, and brain fog in long COVID patients [45,46]. Furthermore, investigations into the involvement of viral infections in the disease and the development of effective antivirals are still required as not only exogenous viruses but also reactivation of human endogenous retroviruses (HERVs) have been reported in some ME/CFS patients [47], opening a new avenue for the development of alternative antiviral therapies targeting HERV as in other diseases [48]. 

### 2.4. Clinical Perspectives

Herpesvirus is considered the main cause of up to 80% of ME/CFS cases, making antivirals a logical treatment option. ACV is primarily active against HSV 1 to 3 but only moderately effective against HSV 4 to 6. VACV is converted to ACV in the liver and offers three to five times the bioavailability after oral ingestion and is, thus, more effective against herpesviruses 4 to 6. It is recommended to start with a high dose (1 g twice daily) for the first ten days as inadequate dosing may cause resistance. The following dose should be 500 mg twice daily provided a normal creatinine clearance. Safety profiles of VACV (≤1000 mg/day), ACV (800 mg/day), and placebo were similar [49]. Extensive sensitivity monitoring has demonstrated a very low rate of resistance [50].

## 3. Immune System Abnormalities

Immunological disturbances have been found in ME/CFS patients, including changes in cytokine profiles [51], increased numbers of B-cell subsets [52,53,54,55], and decreased cytotoxicity of natural killer (NK) and T cells [56,57,58], which play a role in the immune defence against viral infections [59]. Furthermore, an autoimmune aspect of ME/CFS has been implicated, with autoantibodies detected in 4 to 95% of ME/CFS, including antinuclear antibodies, antibodies to neurotransmitters and neurotransmitter receptors, and antibodies formed by oxidative and nitrosative stress (O&NS) (reviewed in [23,60]). Additionally, decreased levels of immunoglobulin G (IgG) subclasses IgG3 and IgG4 have been reported in subsets of patients [61]. Inflammatory, autoimmune, and immunodeficiency problems reported in ME/CFS patients justify the need for treatments targeting these disturbances.

Several CTs targeting each aspect of immune system abnormalities have been investigated within ME/CFS patients with varying levels of success (Table S2).

### 3.1. Inflammation

#### Low-Dose Naltrexone

Naltrexone is a synthetic opioid receptor antagonist utilized in the treatment of opioid addiction [62]. It also binds to non-opioid receptors, such as TLR4 involved in inflammatory-signalling pathways [62]. Low-dose Naltrexone (LDN) has been explored for the treatment of several inflammatory conditions with a high pain burden, such as FM, Crohn’s disease, and multiple sclerosis (MS) [62]. 

A retrospective analysis of 218 ME/CFS patients treated with LDN reported that 73.9% had an improvement in an average of two symptoms [63]. However, improvement was not measured using structured questionnaires or rating scales but was based on patient self-reports. In a case series of three LDN-treated ME/CFS patients, increased energy, improved sleep, and reduced pain were reported in all three patients [64].

### 3.2. Autoimmunity

#### 3.2.1. Immunosuppressive Treatments

In 2009, a cancer patient reported an improvement in their ME/CFS symptoms during chemotherapy, believed to be caused by methotrexate-induced B-cell depletion [65]. The authors suggested the potential benefit of rituximab, a monoclonal CD20 antibody which depletes B lymphocytes, as a treatment for ME/CFS. They trialled rituximab in three ME/CFS patients, all of whom reported symptom improvement. These results suggested B lymphocytes’ involvement in ME/CFS pathogenesis, providing the rationale for undertaking rituximab CTs in ME/CFS [65]. In an RCT, rituximab or saline placebo was administered twice, two weeks apart, in a total 30 ME/CFS patients [66]. There was no significant difference between the treatment and placebo groups at three months postintervention. However, a difference in symptom improvement was observed between the two groups at six to ten months postintervention. A subsequent OPT assessing maintenance doses of rituximab in 29 participants with ME/CFS found similar response rates [67]. In addition, 11 patients were still in remission at the three-year follow-up. These promising results led to a Phase III multicentre RCT in 151 ME/CFS patients [68]. Results demonstrated that repeated treatments with rituximab were not associated with ME/CFS clinical improvement and the authors hypothesized this could be due to only a subgroup of ME/CFS patients having an autoimmune mechanism [68]. To test this hypothesis, future CTs in ME/CFS patients with elevated levels of autoantibodies should be considered. 

Cyclophosphamide is an immunosuppressant utilized in cancer treatment and autoimmune diseases [69]. It has broader immunosuppressive properties compared to rituximab, targeting multiple types of lymphocytes [69]. One study trialled cyclophosphamide in ME/CFS patients in an OPT of 40 ME/CFS patients with a clinical response reported in half of the patients [70]. RCTs are required to confirm the potential benefit of cyclophosphamide in ME/CFS, noting the serious adverse effects of cyclophosphamide, including its association with ovarian failure [69]. 

#### 3.2.2. Immunoadsorption

Immunoadsorption is the removal of plasma antibodies through apheresis and is used in autoimmune diseases to remove pathogenic autoantibodies [71]. A proof-of-concept (POC) study trialled immunoadsorption in ten patients with severe ME/CFS with infection-triggered onset and elevated levels of antibodies against β2-adrenergic acetylcholine receptors [72]. Immunoadsorption reduced β2 IgG antibodies in nine patients, which remained significantly lower six months after treatment compared to baseline. Symptom improvement was reported by seven patients, with long-lasting improvements seen at 6–12 months in three patients. A follow-up study, two years later, retreated five of the patients that had symptom improvements following immunoadsorption, using a modified protocol [73]. Similar outcomes were reported. A recent publication reporting interim results from a POC study trialling immunoadsorption in ten post-COVID ME/CFS patients with elevated β2-adrenergic antibodies found an increase in physical function scores in seven of the patients [74]. 

### 3.3. Immunodeficiency

#### Intravenous Immunoglobulin Treatment

Intravenous immunoglobulin treatment (IVIG) is an antibody preparation used to treat immunodeficient patients and can also be used to suppress autoimmune responses [75]. Four RCTs investigated the efficacy of IVIG for ME/CFS for the improvement of immunological function. Peterson et al., in 1990, reported an improvement in physical and social function, health perception, and mental fatigue in their IVIG group, but this improvement was not significantly different from the placebo group [76]. Another RCT reported 10/23 ME/CFS patients in the IVIG group and 3/26 patients in the placebo group meeting the “responders” criteria of major symptomatic and functional improvement [77]. At a 12-month follow-up, eight of the responders in the IVIG group and all three responders in the placebo group relapsed. An RCT in adolescents with ME/CFS found a significant improvement in functional scores at a six-month follow-up in the IVIG treatment group compared to the placebo group [78]. A larger RCT investigating dose-dependent responses to IVIG treatment in adults with ME/CFS found no association between IVIG dose and symptom improvement, showing no therapeutic benefit of IVIG [79]. 

### 3.4. Future Directions

Although not investigated during a CT, treating ME/CFS patients with LDN appears promising and efficacy should be confirmed in an RCT. Despite the negative outcome from the rituximab Phase III RCT, further RCTs should be considered in a subgroup of ME/CFS patients with elevated autoantibodies. RCTs are also needed to further elucidate the effect of immunoadsorption in ME/CFS, which should also focus on patients with autoantibodies. 

### 3.5. Clinical Perspectives

Immunosuppressive or immunoadsorption therapy currently has no place in daily clinical practice due to expenses and the lack of efficacy in clinical studies. However, blocking the TLR3 with rintatolimod seems promising as do case reports on the aptamer BC007, currently under study in long COVID patients [80]. 

LDN has biological effects that seem to translate into beneficial effects on pain and neuroinflammation in ME/CFS [81]. However, LDN is associated with frequent side effects and slow up-titration to a maximum dose of 4.5 mg is advised and split in a two-dose regimen whereas for patients developing insomnia, a once-daily regimen is recommended.

## 4. Cellular Metabolism Abnormalities

The key symptom of ME/CFS is the presence of persistent fatigue that does not improve with rest. Insufficient energy to meet the body’s demands can lead to both mental and physical fatigue [82]. Mitochondria play a critical role in energy metabolism, producing the energy required by the cell in the form of adenosine triphosphate (ATP). The tricarboxylic acid (TCA) cycle metabolises carbohydrates, amino acids, and fatty acids, regenerating cofactor NADH and providing substrates for ATP synthesis during oxidative phosphorylation (OXPHOS) [83]. 

Given the vital role that mitochondria play in energy metabolism, disturbances may lead to a pathological state, as witnessed in many diseases, including ME/CFS [84,85,86,87,88]. Alterations in mitochondrial morphology, activity, and the levels of intermediary metabolites and enzymes have been reported regardless of disease severity in ME/CFS patients [86]. Specifically, these alterations present as dysregulated glucose and glucogenic amino acid metabolism, impaired provision of substrates for the TCA cycle, impaired OXPHOS, inefficient ATP synthesis, and a shift toward lipid metabolism (reviewed in [86,89]). 

### 4.1. High-Energy Compounds and TCA Cycle Substrates

To counteract the mitochondrial dysfunction found in ME/CFS patients, several treatments have been trialled (Table S3). In an RCT, ME/CFS patients were supplemented with guanidinoacetic acid (GAA), a precursor of creatine naturally occurring in the body [90]. Upon GAA supplementation, creatine levels increased in muscle and serum; however, no effect was observed in general fatigue, exercise performance, or pain, suggesting that creatine availability might not be associated with ME/CFS patient symptomatology [91]. 

Serum levels of acylcarnitine, total carnitine, and free carnitine are decreased in ME/CFS patients in association with symptomatology [92,93]. Reduced carnitine levels affect lipid, protein, and carbohydrate metabolism [94]. Carnitine exists in various forms, including L-carnitine, acylcarnitine (ALC), and propionyl carnitine (PLC) [95]. Supplementation with L-carnitine showed improvements in symptoms and severity in ME/CFS patients [96]. Similarly, in an OPT, ALC or PLC supplementation in ME/CFS patients resulted in significant improvements in cognitive and general fatigue, respectively [97]. A POC study showed a reduction in fatigue levels in 5 of 23 and in 8 of 24 ME/CFS patients after receiving 500 and 1000 mg of anhydrous enol-oxaloacetate three times a day, respectively [98].

### 4.2. Antioxidants

Antioxidants have been extensively studied in ME/CFS. NADH supplementation was used to boost ATP production and antioxidant capacity in two RCT studies in ME/CFS patients, finding symptom improvement and a reduction in maximum heart rate and anxiety during an exercise text [99,100]. Coenzyme Q10 (CoQ10) is another antioxidant, the levels of which are reduced in ME/CFS patients and associated with more fatigue, autonomic symptoms, and cognitive disorders [101]. CoQ10 protects lipid-soluble cell membranes and circulating lipoproteins from oxidative damage by preventing the generation of reactive oxygen species (ROS) during OXPHOS [102]. Moreover, CoQ10 can reduce the activity of inflammatory markers by down-regulating NFκB gene expression [103]. Nutritional supplementation with ubiquinol-10, the reduced form of CoQ10, can reduce fatigue and depression and improve cognitive function and sleep in ME/CFS patients [104]. An RCT combining NADH and CoQ10 supplementation in ME/CFS patients found improved fatigue, quality of life and sleep duration, maximal heart rate, ATP production, and oxidative status [105,106,107]. An OPT combined CoQ10 with selenium supplementation in ME/CFS patients and found it not only improved overall fatigue, quality of life, lipid peroxidation, and antioxidant capacity but also decreased levels of circulating pro-inflammatory cytokines [103,108]. 

Plant-derived antioxidants have been widely used in traditional medicine. Ginsenosides regulate mitochondrial energy metabolism, promoting ATP production and regulating ROS levels [109]. Supplementation with hydroponically grown red ginseng (HRG80) improved energy, sleep, pain, mental clarity, and stamina in ME/CFS patients [110]. 

Quercetin, another plant-derived antioxidant, increases antioxidant capacity by regulating levels of glutathione (GSH), influencing signal transduction pathways to enhance antioxidant activities and removing ROS [111]. It also improves fatigue by reducing muscle damage and increasing fatty acid β-oxidation [112,113,114]. An RCT supplementing quercetin in idiopathic chronic fatigue patients significantly improved fatigue, sleep, and muscle performance [115].

### 4.3. Mitochondrial-Modulating Nutrients

One of the key metabolic disturbances in ME/CFS patients is suboptimal ATP synthesis [7,86]. Supplementation with d-Ribose, a key component of ATP, can restore tissue energy levels following intense exercise in healthy subjects [116]. In an OPT in patients with ME/CFS (n = 9), FM (n = 15), or both ME/CFS and FM (n = 13), d-Ribose supplementation significantly improved energy levels, sleep quality, and pain threshold when analysing all patients grouped together [117]. Positive results were confirmed in an extended cohort of 257 patients (n = 53 ME/CFS, n = 67 FM, and n = 43 ME/CFS + FM) [118]. However, both studies failed to include a control group. 

The efficacy of KPAX002, consisting of a low dosage of methylphenidate hydrochloride and a combination of mitochondrial-modulating nutrients like ALC and selenium, in ME/CFS patients has been trialled in two CTs [119,120]. Interestingly, a POC study in fifteen ME/CFS patients taking KAPX002 reported a significant decrease in fatigue and concentration disturbances [119], whereas a Phase II RCT on 135 ME/CFS subjects reported no significant differences between treatment and placebo groups [120].

### 4.4. Future Directions

In general, treating mitochondrial dysfunction can be effective in addressing the ME/CFS symptoms related to ME/CFS. Except for GAA and KPAX002, supplementation with TCA cycle substrates (L-carnitine, ALC, PLC, oxaloacetate), D-ribose, or antioxidants (NADH, CoQ10, HRG80, quercetin) improved fatigue, exercise performance, mental clarity, pain, and sleep quality. Future RCTs should not only validate the effectiveness of these treatments in a larger group of patients but should also explore the potential benefits of combining multiple supplements.

### 4.5. Clinical Perspectives

Lipid replacement therapy and antioxidants can increase mitochondrial function and reduce fatigue in patients with ME/CFS [121] and even in very high doses, such supplements do not have acute or chronic toxicity [122]. Deficiencies in important antioxidants such as vitamin C and selenium seem secondary to the illness process rather than due to inadequate diets and seem more important in the severity and exacerbation of ME/CFS symptoms [123]. Additional supplements such as 1500 mg of vitamin C and 100 µg of selenium to usual multivitamin supplements seems rational. Further adding vitamin B12 and folic acid could be useful as shown in a cross-sectional survey [124]. 

Supplying CoQ10 200 mg in combination with NADH 20 mg seems to counteract the loss in the effectiveness of the electron transport chain and has shown positive results in a placebo-controlled study [107].

## 5. Gastrointestinal Disturbances

The majority of ME/CFS patients report GI disturbances including nausea, diarrhoea, constipation, abdominal pain, and bloating [125,126]. In an Australian cohort, 28% of patients described a GI-related infectious trigger for their illness and 38% reported having comorbid IBS [127]. IBS comorbidity estimates range from 17 to 92% in ME/CFS patients, versus 10 to 20% in the general population [3]. 

Several studies have shown structural changes in the GI microbiome in ME/CFS, including reduced species diversity and increased heterogeneity amongst ME/CFS patients [128,129,130,131,132,133]. Of note, the microbiome composition in ME/CFS patients with comorbid IBS is distinct from those without [131,132]. Recently, two multi-omics studies found a reduction in short-chain fatty acid (SCFA)-producing bacteria, correlating with symptom severity [131,133]. Both studies replicated earlier findings of reduced *Faecalibacterium* levels, a SCFA-producing bacterium [129,132,134]. SCFAs have anti-inflammatory effects and contribute to intestinal barrier maintenance [135]. Increased bacterial translocation and GI permeability are reported in ME/CFS patients [125,136,137,138,139,140] caused by GI inflammation as part of the ”leaky gut” hypothesis [140,141]. Treatments for GI disturbances trialled in ME/CFS patients aim to restore a healthy microbiome, by either removing pathogenic microbes or by the supplementation of or replacement with beneficial (probiotic) microbes (Table S4).

### 5.1. Antibiotics

Initial culture-based research identified increases in *Enterococcus* and *Streptococcus* spp. in ME/CFS stool samples, leading to the hypothesis that ME/CFS is caused by excessive D-lactic acid production leading to D-lactic acidosis [142]. An OPT evaluating antibiotics in ME/CFS patients with increased stool *Streptococcus* counts reported improved sleep in patients whose stool *Streptococcus* counts decreased after treatment [143]. Neomycin has been trialled in a subset of ME/CFS patients with confirmed small intestinal bacterial overgrowth (SIBO) with patients reporting improvements in pain, depression, and cognitive function [144]. While these preliminary studies reported symptom improvement, it is worth noting the long-term implications of antibiotic use including increased risk of GI, immune, and neurocognitive diseases [145]. 

### 5.2. Probiotics

Probiotics are defined by the World Health Organization (WHO) as “live microorganisms which when administered in adequate amounts confer a health benefit on the host” [146]. Probiotics have the potential to improve GI health and reduce microbiome-related inflammation [147,148] with several studies having investigated probiotics in ME/CFS. In one RCT, patients received *Lactobacillus casei* for eight weeks, which led to an increase in the relative abundance of both *Lactobacillus* and *Bifidobacterium* and a reduction in anxiety [149]. Another RCT found a reduction in inflammation markers in 48 ME/CFS patients administered *Bifidobacterium infantis* 35,264 for eight weeks [150]. While these studies reported improvements, it is important to note that neither study measured core ME/CFS symptoms. An OPT on a probiotic yoghurt containing *Lactobacillus paracasei* ssp. paracasei F19, *Lactobacillus acidophilus* NCFB 1748, and *Bifidobacterium lactis* Bb12 found no significant changes in general health and physical activity [151]. More recently, two additional OPTs were published. One OPT proposed that antibiotics followed by a course of probiotics consisting of *Lactobacillus* and *Bifidobacterium* strains would improve ME/CFS symptoms by reducing D-lactate production (the D-lactate hypothesis) [152]. Improvements in cognition, subjective symptoms, and sleep quality were reported; however, no change in D-lactate was measured, questioning the D-lactate hypothesis. A second study examining the effect of several mixtures of probiotics on general wellbeing, inflammation markers, oxidative stress, and mood in nine ME/CFS patients reported non-significant improvement in symptoms and alterations in inflammatory markers [153]. 

### 5.3. Faecal Microbiota Transplantation (FMT)

FMT is the wholesale transfer of gut microbes from a healthy donor to a patient [154]. This requires careful screening of the donor, followed by processing of the donated stool sample and delivery of the transplant via naso-jejunal tube, gastroscopy, endoscopy, retention enema, or capsules [155]. FMT is widely used in the UK NHS for the treatment of recurrent *Clostridium difficile* infection (rCDI), where it achieves >90% success [156]. Mixed results have been reported for diseases associated with an altered GI microbiota composition, including IBS, IBD, and metabolic disorders [157].

Interest in FMT as a treatment for ME/CFS originates from a retrospective study administering a culture of 13 common faecal bacteria via transcolonic or rectal infusion in 60 ME/CFS patients, most of whom had IBS [158]. After four weeks, 70% of patients responded to the treatment and ME/CFS remained resolved in 58% of contactable patients after 15–20 years. A recent retrospective study found promising results for FMT in ME/CFS, comparing it to an oral treatment consisting of a combination of dietary and lifestyle management, pre- and probiotics, and natural remedies [159]. Patients receiving FMT received ten treatments from ten different donors, and 17/21 achieved at least 60% improvement, with the improvement being significantly higher than that of the oral treatment. However, a small RCT found no improvement in ME/CFS patients after FMT, as measured by subjective the Visual Analogue Scale, the Modified Fatigue Impact Scale, and two health-related quality-of-life scales [160]. A larger Phase II RCT is currently ongoing [161].

### 5.4. Future Directions

Most CTs are OPT; therefore, RCTs should be undertaken, first confirming efficacy in ME/CFS patients with GI disturbances prior to trialling in other ME/CFS patient cohorts. Given the recently discovered reduction in SCFAs in ME/CFS, probiotic formulations including SCFA-producing bacteria should be trialled. 

Results regarding the benefit of FMT in ME/CFS patients are conflicting and need to be confirmed in RCTs in larger cohorts of ME/CFS patients with GI disturbances. Careful consideration needs to be given to the method of delivery, as colonoscopy is an invasive procedure which could negatively impact ME/CFS patients’ symptoms. Capsules are the least invasive method of delivery. In rCDI, capsules are as effective as colonoscopy [162]. Although capsules have received mixed results in treatment of IBS [163,164], FMT efficacy may be improved by pretreatment with antibiotics [165,166] and this combination should be considered for FMT in ME/CFS. 

### 5.5. Clinical Perspectives

Salivary glands exhibit pathological changes in ME/CFS with mast cell accumulation and are also the target for autoantibodies against muscarinic receptors [167]. Deficient saliva production may have important consequences for nutrition and for oral health. Drugs reducing salivary production (primarily antidepressant drugs) may enhance the effect of autoantibodies. Stimulation with the acetylcholinesterase inhibitor pyridostigmine (Mestinon) starting at a dose of 10 mg three times daily or directly with pilocarpine (Salagen) 5–10 mg three times daily is often effective [168]. Nausea and obstipation should be addressed appropriately with available medications. Dietary counselling is important and adjusting the microbiome has great therapeutic potential but needs larger, well-defined studies in ME/CFS [169].

## 6. Neurological Disturbances

The WHO classifies ME/CFS as a neurological disease [170,171] with neurological symptoms including cognitive abnormalities, autonomic dysfunction, sleep disturbances, and altered sensory and pain perception [172]. There have been multiple CTs focused on elucidating the neurological underpinnings of ME/CFS with potential to greatly improve patients’ quality of life (Table S5).

### 6.1. Antidepressants

In the 1990s and early 2000s a number of antidepressants were trialled in patients diagnosed according to the 1994 Fukuda criteria for CFS. Of note, the 1994 Fukuda criteria received criticism for their broad criteria which enabled patients with depression to be misclassified as CFS [173]. 

The monoaminoxidase inhibitors (MAOI) moclobemide and phenelzine have been investigated in ME/CFS patients. Moclobemide increased vitality and energy in patients, rather than decreasing depression [174]. A phenelzine RCT reported no significant improvements [175]. 

An OPT investigating the efficacy of S-citalopram, a selective serotonin reuptake inhibitor (SSRI), in ME/CFS patients (n = 19) with a comorbidity of major depressive disorder demonstrated noteworthy reductions in the symptomatic manifestations of both ME/CFS and depressive symptoms [176]. In an RCT on fluoxetine, another SSRI, neither the depressed nor non-depressed ME/CFS cohorts showed significant improvement [177]. In contrast, an RCT on fluoxetine found improved depressive symptoms but not improved fatigue levels [178].

Duloxetine, a potent serotonin–norepinephrine reuptake inhibitor (SNRI), was investigated for its potential analgesic effects in an RCT [179,180]. While there was no significant difference in general fatigue between the duloxetine group and the placebo group, there was a notable improvement in mental fatigue, pain, and the overall impression of symptom severity among those treated with duloxetine [180]. However, it is important to note that duloxetine-treated patients reported mild to severe adverse effects, including nausea, somnolence, constipation, dizziness, dry mouth, and suicidal ideation.

### 6.2. Psychostimulants

Most pharmaceuticals trialled as psychostimulants in ME/CFS are amphetamines or have amphetamine-like structures and are used for their potential in elevating mood, reducing fatigue, and increasing alertness, concentration, and motivation [181]. Their cardiovascular effects have led them to be used in orthostatic intolerance [182].

Lisdexamfetamine dimesylate (LDX), a psychostimulant used to treat attention-deficit/hyperactivity disorder (ADHD) symptoms, was studied as a potential treatment for cognitive impairment in ME/CFS [183,184]. Of the patients recruited to the RCT, almost 70% had diagnosed or undiagnosed ADHD. Patients reported significant improvements in fatigue, pain, musculoskeletal pain, as well as executive and global functioning after treatment. The precise mechanism of LDX remains unclear, but it is believed to potentially reduce pain by enhancing the filtering of painful stimuli without directly acting as an analgesic [185]. LDX, as a stimulant, affects central dopaminergic and noradrenergic systems in the prefrontal cortex, which may explain the executive functioning improvements observed in ME/CFS patients [186]. This modulation of dopamine circuits also has anti-fatigue effects [187,188]. 

Modafinil induces wakefulness and is a psychostimulant used in sleep disorders [189]. A small RCT of modafinil in ME/CFS patients (n = 14) observed no effects on performance, fatigue, quality of life, or mood [190]. 

### 6.3. Supplementation

A recent study trialled combined supplementation of melatonin and zinc in ME/CFS patients [191]. Patients reported a significant reduction in the perception of physical fatigue and improvements in health-related quality of life; however, no relevant improvements in sleep quality, anxiety, or depression were detected. Treatment withdrawal effects were observed in the medicated cohort, with symptomatic relapse in sleep, physical function, and mental health parameters. There is evidence of melatonin and zinc having positive effects in ME/CFS patients, namely related to fatigue, memory/concentration, motivation, and functional activity [192] as well as depression, cognitive disorders, immune dysfunction, and oxidative stress [193,194].

A recent RCT found that a botanical product containing cistanche and ginkgo extracts improved ME/CFS symptoms [195]. Ginkgo is an antioxidant that improves memory and cognitive deficits and alleviates psychological and physiological distress and fatigue in subjects with mental health disorders [196]. Cistanche is a popular dietary ingredient in Chinese traditional medicine shown to delay the onset of fatigue [197]. An RCT showed a significant improvement in memory, concentration, physical fatigue, unrefreshing sleep, and postexertional malaise (PEM) in both high- and low-dose cohorts [195]. Blood ammonia and lactic acid levels were lower in treated groups compared to healthy controls. Lactic acid is produced and accumulated in muscles under high energy demand and insufficient oxygen supply which is commonly observed in ME/CFS subjects [198]. Ammonia is a waste product of nitrogen-containing compounds, and its circulation and accumulation have a significant impact on fatigue due to its capacity to disturb neuropsychological function and reduce muscle contraction [199].

### 6.4. Fludrocortisone

Neurally mediated hypotension (NMH) is a form of orthostatic intolerance and is witnessed in a subset of ME/CFS patients [200,201]. Fludrocortisone has previously been investigated as a treatment for NMH in ME/CFS patients due to its ability to increase blood volume and pressure [202,203]. Initially, fludrocortisone was investigated during an RCT in ME/CFS patients and no improvements were observed [203]. A subsequent RCT trialled fludrocortisone in ME/CFS patients with confirmed NMH but, again, no clinical benefits were found [202]. 

### 6.5. Future Directions

Alleviating ME/CFS patients’ neurological disturbances remains challenging as the neuropathophysiology of ME/CFS is complex. An ongoing cross-sectional neuroimaging study aims to establish brain signature group differences and develop neuromarkers for diagnosis [204]. A recent systematic review proposes that delayed neurovascular coupling (NVC) and reduced cerebral blood flow lead to oxidative stress, inadequate neuronal energy supply, and neuroinflammation, resulting in subtle and diffuse chronic brain injury [205]. Abnormal NVC has been observed in other brain disorders [206,207] and promoting NVC function has already been explored by increasing global brain blood flow [208], modifying diet [209], modulating calcium channels [209], and transcranial electrical stimulation [209].

Neuroinflammation is another proposed neuropathophysiological mechanism in ME/CFS [210]. High-dose thiamine has demonstrated fatigue reduction in neuroinflammatory conditions, and it has been posited that it may act similarly to carbonic anhydrase inhibitors in reducing intracranial hypertension, which may warrant investigation in a CT for ME/CFS [211,212]. Lastly, given the recognition of mast cells’ involvement in neuroinflammatory processes across various neuroinflammatory brain diseases [213], investigating mast cell stabilisers like H1 and H2 antihistamines, vitamins C and D, and bioflavonoids (e.g., luteolin, quercetin, rutin) with known stabilising effects may be warranted. 

### 6.6. Clinical Perspectives

Many ME/CFS patients have symptoms of autonomic dysfunction partly associated with the production of autoantibodies against the autonomic nervous system [168] and partly because of prolonged bed rest in severe cases. Specific symptoms caused by antibodies, i.e., postural tachycardia, hyperactive bladder dysfunction, nausea due to spasm of the pyloric sphincter, reduced salivary gland function, and obstipation should be addressed appropriately with available medications. Deconditioning could be avoided by intermittent exposure to a sitting or upright posture or compensated by adding salt 1 g/litre of fluid and/or by desmopressin 60–120 µg daily [214]. Counteracting the down-regulation of the renin–aldosterone system by implementing daily doses of fludrocortisone 0.1 mg has not been shown to be ineffective [202] but information on higher doses used in other patients groups is not available. 

## 7. Neuroendocrine Disturbances

The suppression of neuroendocrine axes described for ME/CFS is characterized by a reduction in hormone production, due to the influence of pro-inflammatory cytokines and O&NS [215,216,217]. ME/CFS patients present dysregulation of the following neuroendocrine axes:

- Thyrotropic (HPT) axis: low thyroid hormone activity, which is linked to fatigue [218,219];
- Somatotropic (HPS) axis: deficient growth hormone (GH) regulation [220], with loss of pulsatile function of GH secretion in response to exercise [221], resulting in loss of muscle and bone mass, muscle weakness, and changes in glucose and fat metabolism;
- Adreno-cortical (HPA) axis: mild hypocortisolism, lack of pulsatile stimulus leading to adrenal atrophy and a heightened negative feedback loop [222];
- Gonadotropic (HPG) axis: abnormalities in gonadal hormones lead to earlier onset of menopause [223] and reduced oestrogen receptor levels in the immune system [224].

### 7.1. Treatments with Peripheral Hormones

Direct supplementation of deficient hormones has been explored in ME/CFS patients (Table S6). Replacement of cortisol, a hormone produced by the adrenal gland, using hydrocortisone was explored during an RCT following the finding of HPA axis inactivation in ME/CFS patients [225]. Hydrocortisone was administered orally in the morning (20–30 mg) and afternoon (5 mg) to mimic the diurnal fluctuation of normal cortisol levels. Although a significant improvement in wellness was reported, there was also significant adrenal suppression, leading authors to conclude that hydrocortisone is not an appropriate treatment for ME/CFS. A subsequent small crossover study found that administering lower dosages (5–10 mg) of hydrocortisone did not suppress adrenal function and patients had improved fatigue and disability levels [226].

Dehydroepiandrosterone (DHEA) is produced by the adrenal gland in response to stress [227]. In a prospective, uncontrolled study administering DHEA to ME/CFS patients with suboptimal DHEA levels in the blood, fatigue, pain, cognitive, and anxiety symptoms were improved following intervention [228]. 

A preliminary study administering GH to ME/CFS patients, targeting the HPS axis, found no notable improvements in their outcome measures, despite patients reporting improvements in symptoms [229]. However, administration of GH has shown positive results in FM, reducing pain and improving patients’ quality of life [230,231,232], but higher concentrations were associated with increased patient mortality in critical illness [233,234]. 

### 7.2. Targeted Therapy

CT38 is a recently developed peptide that selectively down-regulates corticotropin-releasing factor receptor 2 (CRF2) via agonist-mediated receptor endocytosis, restoring the loss of homeostatic control due to CRF2 upregulations in the raphe nuclei and limbic system [235]. A pilot study trialled CT38 in ME/CFS patients (n = 14) based on the hypothesis that ME/CFS abnormalities originate from a single pathway involving CRF2 [235]. Preliminary results show promise with improved symptoms and mild adverse events. However, CT38 is still classified as an experimental drug and the mechanism of action of CT38 requires further validation. 

### 7.3. Future Directions

To date, few CTs have addressed neuroendocrine dysfunction in ME/CFS (Table S6). Multiple therapeutic approaches have been proposed for neuroendocrine disturbances in ME/CFS patients, based on findings in FM and critical illness (reviewed in [236]). Suggestions include the administration of multiple peripheral hormones simultaneously due to the complementary roles of some hormones. In the context of ME/CFS, some clinicians recommend a combination of thyroid, adrenal, and gonadal hormones [237]. 

Alternate suggestions to overcome the negative feedback loops and toxic overdoses associated with administering peripheral hormones include treatments aiming to reactivate the central endocrine glands, using tropic hormones or other secretagogues [236]. Interventions based on the “bi-stability model” have also been proposed in ME/CFS, aiming to reset the HPA axis by shifting patients to a “normal-cortisol steady-state” [238,239].

Additionally, a subgroup of hypothyroidic ME/CFS patients have recently been identified with autoimmunity to the selenium transporter SELENOP [240]. A combined administration of selenium and triiodothyronine (T3) has been proposed as a potential treatment for this subset of patients and provides a potential therapeutic opportunity for future CTs.

### 7.4. Clinical Perspectives

In clinical practice, it is common to measure thyrotropin (TSH) and only measure levels of total thyroxine (TT4) and triiodothyronine (TT3). However, a case-controlled study showed that ME/CFS patients have lower levels of free T3 despite similar TSH levels and higher levels of reverse T3, indicating depressed tissue levels of T3 [219]. An in-depth analysis of thyroid function followed by appropriate substitution seems reasonable. Pituitary–adrenal abnormalities may produce chronic fatigue. Studies in the broader ME/CFS patient group have detected relative hypocortisolism and altered dynamic responses [241], and in the clinical setting, many ME/CFS patients have symptoms compatible with a disturbed diurnal rhythm and treatment with low-dose hydrocortisone (5–10 mg) in the morning seems useful without an associated risk of supressing adrenal function [226].

## 8. Outcome Measures Used in ME/CFS Research

### 8.1. Patient-Reported Outcome Measures

Outcome measures are essential in intervention trials to determine whether there is a clinically significant improvement in response to the intervention [242]. In ME/CFS research, 96% of RCTs use subjective patient-reported outcome measures (PROMs) as their primary outcome measure [243]. PROMs are questionnaires completed by patients to measure the subjective elements of their condition, such as pain intensity and activity limitations and health-related quality of life [244]. There are different types of PROMs, including generic, disease-specific, and domain (symptom)-specific [245].

It is important to be mindful of the limitations associated with the use of PROMs. All PROMs are subjective and susceptible to inter-individual variability and bias [244]. In the context of ME/CFS, heterogenous clinical presentation could increase inter-individual variability. Furthermore, recall bias is a particular concern due to ME/CFS patients commonly experiencing cognitive impairment [246], demonstrated by a weak correlation between subjective and objective measures of activity levels [247]. In addition, PROMs are susceptible to ceiling and floor effects, where participants score the maximum and minimum score, respectively. This poses an issue in CTs as patients with a clinically significant improvement following treatment may still report the maximum score on PROMs [248]. There is the risk of overestimating treatment response with PROMs, particularly in open-label settings [244]. This has been demonstrated in ME/CFS patients during the KPAX002 CTs. A significant improvement was reported using PROMs in a POC study [119], which was later disputed by an RCT reporting no improvement using the same PROMs [120]. Furthermore, generic PROMs are less sensitive to changes in a patient’s condition [249]. 

Due to these limitations, the reliability and validity of PROMs should be confirmed in patient cohorts prior to their use in CTs [250]. Reliability refers to the degree to which a measure consistently produces identical scores when administered multiple times, assuming the patient’s condition remains stable. Validity is the measure of how well an instrument assesses the concept it was designed for [250]. 

The 36-item short-form health survey (SF-36), the checklist individual strength (CIS), and the Chalder Fatigue Questionnaire (CFQ) are common frequently used PROMs in ME [243]. The SF-36 is a generic PROM designed to measure health-related quality of life [251], whereas the CIS [252] and the CFQ [253] are both domain-specific PROMs designed to measure fatigue, with the former being specifically developed for ME/CFS. These PROMs were found to be reliable and valid measures in the general population, but evidence for their suitability for use in a ME/CFS population is limited [254]. Although there is evidence that the SF-36, CIS, and CFQ can distinguish patients from controls [248,252,255], they are unable to distinguish between ME/CFS and other patient populations such as MS, lupus, depression, and idiopathic chronic fatigue [255,256,257,258]. This questions their sensitivity for ME/CFS. Indeed, high ceiling effects in ME/CFS patients were reported for both the SF-36 [248,259] and the CFQ [260]. Finally, test–retest reliability was poor for the SF-36 [261], the CFQ had only been tested in 15 ME/CFS patients with an undefined retest period [262], and the CIS has high test–retest reliability in the general population [263] but has not been tested in ME/CFS patients. 

We, therefore, recommend additional investigations into the validation and reliability of these measures in a larger cohort of ME/CFS patients to confirm their suitability. Once validated, PROMs should only be used to complement objective measures and clinical assessments to give a full overview of treatment efficacy or when an appropriate objective measure does not exist. 

### 8.2. Objective Outcome Measures 

Due to the aforementioned limitations of using PROMs in ME/CFS CTs, researchers are focussed on identifying suitable objective measures for ME/CFS patients. Objective measures of exertion intolerance, the cardinal symptom of ME/CFS, have been assessed. These include accelerometers to monitor activity levels [247,264,265,266,267,268,269,270], cardiopulmonary exercise testing to characterize exercise performance [269,271], and the hand grip strength test to measure muscle fatigability [272,273]. Objective measures of sleep defects (reviewed in [274]) and cognitive impairment (reviewed in [275]) have also been assessed in ME/CFS patients. 

Accelerometers collect data on types of activity, intensity, posture, total volume of physical activity, and total energy expenditure [276]. Validation studies concluded that step count has the best accuracy in measuring overall activity levels [277]. Indeed, step count was significantly different between mild, moderate, and severe ME/CFS patients [269]. However, as well as step count, metabolic equivalents (METs), total energy expenditure, and time spent active were also able to distinguish ME/CFS patients from sedentary controls [267]. These other measures may be more suitable for ME/CFS patients, considering that most severe ME/CFS patients are bedbound. Researchers should use waterproof accelerometers, as for some ME/CFS patients, bathing is the only activity they are able to do within a day. Other considerations have to be made when using accelerometers in clinical studies that include the length of time an activity is recorded and body placement of the accelerometer [276]. As 30% of ME/CFS patients’ activity levels follow a “boom and bust cycle” [278], researchers should ensure activity levels are recorded for a sufficient length of time to capture fluctuations in activity levels due to PEM.

CPETs combine measurements of cardiovascular (heart rate and blood pressure) and respiratory function (oxygen consumed and carbon dioxide produced) during physical exertion using a cycle ergometer or treadmill [279]. Oxygen consumption at peak effort (VO_2_peak) and at ventilatory anaerobic threshold (VAT) are reliable objective measures of maximum energy producing capacity and capacity to do continuous work, respectively [280]. VO_2_peak was significantly lower in ME/CFS patients compared to active and sedentary controls and idiopathic chronic fatigue controls, although some studies found no differences [281,282,283,284,285,286,287]. In healthy adults and some diseases, test–retest measures of oxygen consumption were stable when repeated on consecutive days [288,289,290]. In contrast, oxygen consumption and workload were significantly reduced in ME/CFS patients during a second CPET compared to the first CPET undertaken 24 hours earlier [271,285,286,287,288,291], due to the first CPET inducing PEM [280]. Therefore, the extent of reduced VO_2_peak and VAT is a quantifiable measure of the degree of impaired recovery mechanisms due to PEM and could be used to measure changes in PEM following an intervention. However, there are limitations surrounding the use of CPET. Firstly, there are safety concerns due to the likelihood of exacerbating pain and symptoms in ME/CFS patients [287,292]. Furthermore, it takes ME/CFS patients between 1 and 64 days to recover from a two-day CPET [293]. As PEM affects CPET measures, and patients have to travel to the test site and experience prolonged waiting times, which are both known to exacerbate PEM, the accuracy of CPET in capturing the baseline in ME/CFS patients is questionable [280]. Finally, although CPET has been measured in mild, moderate, and severe ME/CFS patients and significant differences have been found across the disease severities [268,271], CPET is not appropriate for immobile patients. Therefore, due to these limitations, along with the finding of a strong correlation between CPET measures and accelerometery measures [269], we recommend the use of accelerometers as objective measures of functional capacity in ME/CFS patients. 

## 9. Conclusions

In this review, we have discussed published CTs in ME/CFS that have occurred over the past 33 years targeting immunological, metabolic, GI, neurological, and neuroendocrine disturbances in ME/CFS patients. Rintatolimod is the standout and the only current example of a successful treatment for ME/CFS. Yet, its status as an experimental drug means it cannot be incorporated into routine treatment. Other interventions including the use of the antivirals VACV, VGVC, and artesunate, metabolic supplements, and probiotics have shown promising results but need to demonstrate clinical efficacy in larger RCTs. Interventions that have proven effective in or are currently being trialled in other chronic diseases with overlapping symptomology to ME/CFS, such as luteolin in long COVID and mast cell stabilisers in neuroinflammatory disease, could be considered for repurposing in ME/CFS. Future CTs in ME/CFS should include suitable objective outcome measures, such as the use of accelerometers to measure physical activity, and rely less on subjective and often patient based questionnaires. Other important considerations in designing CTs in ME/CFS include having appropriately powered studies and numbers of trial participants that are of a uniform clinical subtype that utilize stringent case definitions that include PEM as a core symptom and use evidence-based inclusion criteria.

## Data Availability

No new data were created or analysed in this review. Data sharing is, therefore, not applicable to this article.

## Supplementary Materials

The following supporting information can be downloaded at: https://www.mdpi.com/article/10.3390/jcm13020325/s1. Table S1: Clinical trials investigating treatments for chronic infections in ME/CFS; Table S2: Clinical trials investigating treatments for immune disturbances in ME/CFS; Table S3: Clinical trials investigating treatments for metabolic disturbances in ME/CFS; Table S4: Clinical trials investigating treatments for gastrointestinal disturbances in ME/CFS; Table S5: Clinical trials investigating treatments for neurological disturbances in ME/CFS; Table S6: Clinical trials investigating treatments for neuroendocrine disturbances in ME/CFS.
