# Supplementary material for: Advancing Research and Treatment: An Overview of Clinical Trials in Myalgic Encephalomyelitis/Chronic Fatigue Syndrome (ME/CFS) and Future Perspectives
Source: J Clin Med. 2024 Jan 6;13(2):325. doi: 10.3390/jcm13020325 (PMC10816159; doi:10.3390/jcm13020325)
Supplement: Supplementary file 1 [file jcm-13-00325-s001.zip › jcm-2776138-supplementary.pdf]

Supplementary Table S1: Clinical trials investigating treatments for chronic infections in ME/CFS.

Intervention (trial code)	Study design (Cohort size)	Diagnostic criteria	Patient subgroup	Treatment regime	Outcome measures	Outcome significance	Ref
Rintatolimod (n/a)	OPT phase I (15)	1994 CDC	Illness severity (KPS 20-60)	400 mg/week for a variable period of time followed by 1200 mg/week 6 months	**PROMs**<br>1) Functional impairment (KPS)<br>2) Perceived cognitive performance (SCL-90-R)<br><br>**Objective measures**<br>1) Exercise tolerance (treadmill)<br><br>**Biological endpoints**<br>1) HHV-6 reactivation	**PROMs**<br>1) $p<0.01$<br>2) $p<0.01$<br><br>**Objective measures**<br>1) $p<0.01$<br><br>**Biological endpoints**<br>1) $p<0.001$	[40]
Rintatolimod (n/a)	RCT (PA) phase II (Rintatolimod: 47, Placebo: 42)	1994 CDC	Illness severity (KPS 20-60), diagnosis >12 months	800 mg/week for 6 months	**PROMs**<br>1) Functional impairment (KPS)<br>2) Perceived cognitive performance (SCL-90-R)<br>3) Daily activity (ADL)<br>4) Psychiatric morbidity (DIS)<br><br>**Objective measures**<br>1) Brain MRI<br>2) Exercise tolerance (treadmill)<br><br>**Biological endpoints**<br>1) HHV-6 reactivation	**PROMs**<br>1) $p<0.05$<br>2) $p<0.05$<br>3) N.S.<br>4) N.S.<br><br>**Objective measures**<br>1) N.S.<br>2) $p<0.001$<br><br>**Biological endpoints**<br>1) N.S.	[41]
Rintatolimod (NCT00215800)	RCT (PA) phase III (Rintatolimod: 93, Placebo: 101)	1994 CDC, 1988 CDC	KPS: 40-60, negative anti dsDNA, diagnosis >12 months	400 mg/week for 2 weeks, then 800 mg/week up to 40 weeks	**PROMs**<br>1) Functional impairment (KPS)<br>2) Daily activity (ADL)<br>3) Vitality (SF-36 subscale)<br>4) General health status (SF-36 subscale)<br><br>**Objective measures**<br>1) Exercise tolerance (treadmill)*	**PROMs**<br>1) $p<0.01$<br>2) N.S.<br>3) N.S.<br>4) $p<0.01$<br><br>**Objective measures**<br>1) $p<0.05$	[42]

VACV<br>(n/a)	RCT (PA)<br>(VACV: 14,<br>Placebo: 13)	1994 CDC	Illness severity<br>(EI≤5),<br>elevated EBV serum IgM against VCA and/or EA detected	Group 1: VACV 4g/day for 6 months or placebo<br><br>Group 2†: Cimetidine 500 mg twice per day or probenecid 500 mg twice per day plus VACV 4g/day	**PROMs**<br>1) Physical functional capacity (EI)<br><br>**Biological endpoints**<br>1) EBV VCA IgM<br>2) EBV EA	**PROMs**<br>1) n/a<br><br>**Biological endpoints**<br>1) n/a<br>2) n/a	[30]
VGCV<br>(n/a)	OPT<br>(12)	1994 CDC	Suspected viral onset	1800 mg/day for 3 weeks then 900 mg/day until 6 months	**Biological endpoints**<br>1) EBV VCA IgG and IgM antibodies<br>2) Antibodies against EBNA<br>3) Antibodies against EBV EA<br>4) HHV-6 IgG and IgM antibodies<br>5) HCMV IgG and IgM antibodies	**Biological endpoints**<br>1) *p*=0.008‡<br>2) N.S.‡<br>3) N.S.‡<br>4) N.S.‡<br>5) N.S.‡	[32]
VGCV<br>(NCT00478465)	RCT (PA)<br>(VGCV: 20, Placebo: 10)	1994 CDC	Suspected viral onset,<br>elevated antibody titers against HHV-6 and EBV	1800 mg/day for 3 weeks and then 900 mg/day until 6 months	**PROMs**<br>1) Fatigue (MFI-20*)<br>2) Mental fatigue (MFI-20 subscale)<br>3) General fatigue, physical fatigue, reduced activity, reduced motivation (MFI-20 subscales)<br>4) Fatigue (FSS)<br>5) symptom severity (CDC-SI)<br>6) Physical functioning (self-report)<br>7) Cognitive functioning (self-report)<br>8) Sleep (SAQ)<br>9) Depression (HAM-D)<br><br>**Objective measures**<br>1) Cognitive function (PASAT)	**PROMs**<br>1) N.S.<br>2) *p*=0.039<br>3) N.S.<br>4) *p*=0.006<br>5) N.S.<br>6) N.S.<br>7) *p*=0.025<br>8) N.S.<br>9) N.S.<br><br>**Objective measures**<br>1) N.S.	[35]

**Biological endpoints**	**Biological endpoints**
1) Monocyte and neutrophil counts	1) $p<0.05$
2) Cytokines	2) $p<0.05$
3) EBV and HHV-6 IgG antibody titers	3) N.S.

Abbreviations:

1988 CDC: 1988 Centers for Disease Control and Prevention criteria for CFS by Holmes, 1994 CDC: 1994 CDC criteria for CFS by Fukuda, CDC-SI: CDC symptom inventory scores, DIS: diagnostic interview schedule, dsDNA: double-stranded deoxyribonucleic acid, EA: early antigens, EBV: Epstein Barr virus, EBNA: EBV nuclear antigens, EI: energy index, FSS: fatigue severity scale, HAM-D: Hamilton depression rating scale, HCMV: human cytomegalovirus, HHV-6: human herpesvirus-6, IgG: immunoglobulin G, IgM: immunoglobulin M, KPS: Karnofsky performance scale, MFI-20: 20-item multidimensional fatigue inventory, MRI: magnetic resonance imaging, n/a: not available, N.S.: not significant, OPT: open-label pilot study, PA: parallel arm, PASAT: paced auditory serial addition test, PROMs: patient-reported outcome measures, RCT: randomized placebo-controlled double-blinded clinical trial, SAQ: sleep assessment questionnaire, SCL-90-R: symptom checklist 90-R, VACV: valacyclovir, VGCV: valganciclovir, VCA: viral capsid antigen.

Trial codes:

NCT: ClinicalTrials.gov

Footnotes:

* Marks a primary outcome measure

† After 3 months of VCV, if the EI point score did not improve, participants from group 1 were switched to group 2.

‡ *P* values reported for “responders”

Supplementary Table S2: Clinical trials investigating treatments for immune disturbances in ME/CFS.

Intervention (trial code)	Study design (Cohort size)	Diagnostic criteria	Patient subgroup	Treatment regime	Outcome measures	Outcome significance	Ref
**Autoimmunity**							
Cyclophosphamide (NCT02444091)	OPT phase II (40)	CCC	Disease duration >2 years	6 infusions (first infusion 600 mg/m^2 followed by 700 mg/m^2) at monthly intervals	**PROMs**<br>1) Fatigue*†<br>2) Functional level†<br>3) Vitality, social function, bodily pain (SF-36 subscales)<br>4) Fatigue (FSS)<br>5) Physical function (SF-36 subscale)<br>6) Physical activity (SF-36-PCS)<br><br>**Objective measures**<br>1) Physical activity (number of steps)	**PROMs**<br>1) N.S. (3 months); *p*<0.001 (6, 9, 12, 15, 18 months)‡<br>2) N.S. (3 months); *p*<0.01 (6 months); *p*<0.001 (9, 12, 15, 18 months)<br>3) n/a<br>4) n/a<br>5) *p*<0.001 (3, 6, 9, 12, 15, 18 months)<br>6) n/a<br><br>**Objective measures**<br>1) *p*<0.01 (17-18 months), *p*<0.001 (7-9, 11-12 months)	[70]
Immunoadsorption(n/a)	POC (10)	CCC	Infection-triggered onset,<br>severity (Bell scale of <50),<br>elevated ß2 antibodies	5 cycles of immunoadsorption at days 1-3 and 6-7. After the 5th cycle patients received 25g IV IgG	**PROMs**<br>1) Symptom severity†<br>2) Fatigue and cognitive impairment (FACT-F)<br><br>**Objective measures**<br>1) Muscle strength (pinch dynamometer)<br>2) Physical activity (steps/day)<br><br>**Biological endpoints**<br>1) ß1 and ß2 IgG<br>2) M3/M4 receptor IgG<br>3) Total serum IgG<br>4) Tetanus IgG and PcP IgG<br>5) Endothelial function (PAT)	**PROMs**<br>1) n/a<br>2) *p*=0.038 (3 months), *p*=0.045 (6 months)<br><br>**Objective measures**<br>1) N.S.<br>2) N.S.<br><br>**Biological endpoints**<br>1) N.S. (3 months), *p*<0.05 (6 months)<br>2) N.S.<br>3) N.S.<br>4) N.S.<br>5) N.S.	[72]

Rituximab<br>(NCT00848692)	RCT (PA) phase II<br>(Rituximab: 15, Placebo: 15)	1994 CDC	-	Treatment: 2 infusions of 500 mg/m^2, 2 weeks apart<br><br>Placebo: 2 infusions of equal volume of saline, 2 weeks apart	**PROMs**<br>1) Fatigue severity at 3 months post intervention*†<br>2) Fatigue severity†<br>3) Symptom severity†<br>4) Physical health (SF-36 subscale)<br>5) Physical function (SF-36 subscale)<br>6) Bodily pain (SF-36 subscale)<br>7) General health, social function, role emotional, mental health (SF-36 subscales)<br><br>**Other**<br>1) Physician assessed fatigue severity	**PROMs**<br>1) N.S.<br>2) *p*=0.045<br>3) n/a<br>4) *p*=0.039<br>5) *p*=0.014<br>6) *p*=0.005<br>7) N.S.<br><br>**Other**<br>1) *p*=0.021	[66]
Rituximab<br>(NCT01156909)	OPT phase II<br>(28)	1994 CDC<br>CCC	-	2 infusions of 500 mg/m^2, 2 weeks apart<br>Maintenance infusions at 3, 6, 10, and 15 months	**PROMs**<br>1) Fatigue severity†<br>2) Health related quality of life (SF-36)	**PROMs**<br>1) n/a§<br>2) N.S. (3 months); *p*<0.01 (15 months); *p*<0.001 (6, 10, 20, 30, 36 months); *p*<0.0001 (24 months)	[67]
Rituximab<br>(NCT02229942)	RCT (PA)<br>(Rituximab: 77, Placebo: 74)	CCC	Disease duration 2-15 years (or ≥5 years if the disease was mild)	2 infusions of 500 mg/m^2 Rituximab, 2 weeks apart<br>Maintenance infusions at 3, 6, 9, and 12 months	**PROMs**<br>1) Fatigue severity†<br>2) Health related quality of life (SF-36)<br>3) Fatigue severity (FSS)<br><br>**Objective measures**<br>1) Physical activity (number of steps)	**PROMs**<br>1) N.S.<br>2) N.S.<br>3) N.S.<br><br>**Objective measures**<br>1) N.S.	[68]
**Immunodeficiency**							
IVIG<br>(n/a)	RCT (PA)<br>(IVIG: 23, Placebo: 26)	1988 CDC	-	3 doses of IVIG (2g/kg/month)<br><br>Placebo: 10% w/v maltose in equivalent volume	**PROMs**<br>1) Physical wellbeing (QAL)<br>2) Depression (Hamilton score)<br>3) Depression (Zung scale)	**PROMs**<br>1) *p*<0.01<br>2) *p*<0.01<br>3) n/a	[77]

					**Biological endpoints**<br>1) T cell analysis<br>2) DTH skin testing<br><br>**Other**<br>1) Physician assessment of symptomatic and functional improvement	**Biological endpoints**<br>1) *p*<0.01<br>2) *p*<0.01<br><br>**Other**<br>1) *p*=0.03	
IVIG<br>(n/a)	RCT (PA)<br>(IVIG: 14, Placebo: 14)	1988 CDC	-	6 doses of IVIG (1g/kg/month)<br><br>Placebo: 1% albumin solution	**PROMs**<br>1) Symptom severity†<br>2) Physical and social functioning, health perceptions, and mental health (SF-36 subscales)<br><br>**Biological endpoints**<br>1) IgG subclass	**PROMs**<br>1) N.S.<br>2) N.S.<br><br>**Biological endpoints**<br>1) N.S.	[76]
IVIG<br>(n/a)	RCT (PA)<br>(0.5g/kg IVIG: 22, 1g/kg IVIG: 28, 2g/kg IVIG: 23, placebo: 26)	1992 NIH	-	3 doses of IVIG, participants randomly allocated to receive 0.5 g/kg/month, 1 g/kg/month or 2 g/kg/month)<br><br>Placebo: 1% albumin solution	**PROMs**<br>1) Physical functioning (QAL)<br>2) Functional activity¶<br>3) Mood (POMS)<br>4) Functional impairment (KPS)<br><br>**Biological endpoints**<br>1) DTH skin testing<br>2) T cell analysis	**PROMs**<br>1) N.S.<br>2) N.S.<br>3) N.S.<br>4) N.S.<br><br>**Biological endpoints**<br>1) N.S.<br>2) N.S.	[79]
Immunoglobulin G<br>(n/a)	RCT (PA)<br>(IVIG: 36, placebo: 34)	1994 CDC	Adolescents (11-18 years old)	3 doses (1 g/kg/month)<br><br>Placebo: 10% w/v maltose solution with 1% albumin of equivalent volume for weight	**PROMs**<br>1) Anxiety (STAI)<br>2) Depression (BDI)<br>3) General health questionnaire<br><br>**Biological endpoints**<br>1) DTH skin testing<br>2) IgG subclass<br><br>**Other**<br>1) Functional improvement (physician	**PROMs**<br>1) *p*=0.01<br>2) *p*=0.002<br>3) *p*<0.001<br><br>**Biological endpoints**<br>1) N.S.<br>2) N.S.<br><br>**Other**<br>1) *p*<0.04	[78]

			assessed)	

Abbreviations:
1988 CDC: 1988 Centers for Disease Control and Prevention criteria for CFS by Holmes, 1992 NIH: 1992 NIH criteria for CFS by Schluederberg, 1994 CDC: 1994 CDC criteria for CFS by Fukuda, ß1: beta-1 adrenergic receptors, ß2: beta-2 adrenergic receptors, BDI: beck depression inventory, CCC: Canadian consensus criteria for ME/CFS by Carruthers, DTH: delayed-type hypersensitivity, FACT-F: 13 item questionnaire assessing fatigue, FSS: fatigue severity scale, IgG: immunoglobulin G, IV: intravenous, IVIG: intravenous immunoglobulin therapy, M3/M4: muscarinic M3 and M4 acetylcholine receptors, OPT: open-label pilot study, PA: parallel arm, PAT: pulse arterial tonometry, PcP: pneumococcal polysaccharide, POC: proof of concept, POMS: profile of mood states, PROMs: patient-reported outcome measures, QAL: quality of life scale, RCT: randomized placebo-controlled double-blinded clinical trial, SF-36: 36-item short form health survey, SF-36-PCS: SF-36 - physical component summary, STAI: state-trait anxiety inventory.
Trial codes:
NCT: ClinicalTrials.gov
Footnotes:
* Marks a primary outcome measure
† Assessment using unpublished questionnaires created by the authors
‡ *p* values reported for “responders”
§ *p* values not reported, but 64% of participants were said to have a “clinical response” based on fatigue severity scores
¶ Functional activity defined as the hours of self-reported non-sedentary activity per day

Supplementary Table S3: Clinical trials investigating treatments for metabolic disturbances in ME/CFS.

**Intervention (Trial code)**	**Study design (Cohort size)**	**Diagnostic criteria**	**Patient subgroup**	**Treatment regime**	**Outcome measures**	**Outcome significance**	**Reference**
ALC + PLC<br>(n/a)	OPT<br>(ALC: 29, PLC: 30, ALC + PLC: 30)	1994 CDC	-	ALC group: 2 g/day<br>PLC group: 2 g/day<br>ALC+PLC group: 2g/day ALC and 2g/day PLC,<br>6 months	**PROMs**<br>1) Health impression (CGI)<br>2) Fatigue (MFI-20)<br>3) Pain (MPQ-DLV)<br>**Objective measures**<br>1) Stroop test for attention concentration<br>**Biological endpoints**<br>1) Free carnitine and carnitine esters	**PROMs**<br>1) ALC $p<0.0001$<br>PLC $p<0.0001$<br>ALC + PLC N.S.<br>2) ALC $p=0.015$<br>PLC N.S<br>ALC + PLC N.S.<br>3) ALC $p=0.840$<br>PLC $p=0.380$<br>ALC + PLC $p=0.877$<br>**Objective measures**<br>1) ALC $p<0.0001$<br>PLC $p=0.011$<br>ALC + PLC $p=0.004$<br>**Biological endpoints**<br>1) ALC $p<0.05$<br>PLC $p<0.05$<br>ALC + PLC $p<0.05$	[97]
CoQ10 + NADH<br>(NCT02063126)	RCT (PA)<br>(CoQ10 + NADH: 39, Placebo: 34)	1994 CDC	-	CoQ10: 200mg/day<br>NADH 20 mg/day<br>2 months	**PROMs**<br>1) Fatigue (FIS)<br>2) Pain (MPQ)<br>3) Sleep Quality (PSQI)<br>**Biological endpoints**<br>1) Intracellular NAD+/NADH<br>2) CoQ10 levels<br>3) Lipid peroxidation<br>4) ATP production<br>5) Citrate synthase activity<br>**Objective measures**<br>1) Max HR changes	**PROMs**<br>1) $p<0.05$<br>2) N.S.<br>3) N.S.<br>**Biological endpoints**<br>1) $p<0.001$<br>2) $p<0.05$<br>3) $p<0.05$<br>4) $p<0.05$<br>5) $p<0.05$<br>**Objective measures**<br>1) $p<0.05$	[105, 106]

					2) Exercise performance (VO_2, VCO_2)<br>3) Blood pressure (BP)	2) N.S.<br>3) N.S.	
CoQ10 + NADH<br>(NCT03186027)	RCT (PA)<br>(CoQ10 + NADH: 72, Placebo: 72)	1994 CDC	-	CoQ10: 200mg/day<br>NADH 20mg/day<br>3 months	**PROMs**<br>1) Fatigue (FIS)<br>2) Sleep Quality (PSQI)<br>3) Health-related quality of life (SF-36)	**PROMs**<br>1) *p*=0.022<br>2) *p*=0.018<br>3) N.S.	[107]
CoQ10 + Se<br>(NCT05128292)	OPT<br>(30)	1994 CDC	-	CoQ10: 400 mg/day<br>Se: 200 µg/day<br>2 months	**PROMs**<br>1) Fatigue (FIS)<br>2) Sleep Quality (PSQI)<br>3) Health-related quality of life (SF-36)<br><br>**Biological endpoints**<br>1) Total antioxidant capacity<br>2) Lipid peroxidation<br>3) Inflammatory response (IL-1β, IL-6, IL-8, IL-10, TNF-α, and CRP)<br>4) Cardiovascular dysfunction (FGF-21 and NT-proBNP)	**PROMs**<br>1) *p*=0.021<br>2) N.S.<br>3) *p*=0.002<br><br>**Biological endpoints**<br>1) *p*<0.0001<br>2) *p*<0.0001<br>3) *p*<0.01<br>4) N.S.	[108]
D-ribose<br>(n/a)	OPT<br>(36)	1994 CDC (CFS)<br>ACR (FMS)	CFS and/or FMS patients	D-ribose TID 5 g/day<br>3 weeks	**PROMs**<br>1) Energy level (DVAS)<br>2) Sleep (DVAS)<br>3) Mental clarity (DVAS)<br>4) Pain (DVAS)<br>5) Well-being (DVAS)	**PROMs†**<br>1) *p*<0.0001<br>2) *p*=0.0001<br>3) *p*=0.003<br>4) *p*=0.026<br>5) *p*<0.0001	[117]
D-ribose<br>(NCT01108549)	OPT<br>(226)	1994 CDC (CFS)<br>ACR (FMS)	CFS and/or FMS patients	D-ribose TID 5 g/day<br>3 weeks	**PROMs**<br>1) Energy level (DVAS)<br>2) Sleep (DVAS)	**PROMs†**<br>1) *p*<0.0001<br>2) *p*<0.0001	[118]

					3) Mental clarity (DVAS)<br>4) Pain (DVAS)<br>5) Well-being (DVAS)	3) *p*<0.0001<br>4) *p*<0.0001<br>5) *p*<0.0001	
GAA<br>(NCT02213679)	RCT (CO)<br>(21)	1994 CDC	-	GAA: 2.4 g/day<br>3 months	**PROMs**<br>1) Fatigue (FIS)<br>2) Health-related quality of life (SF-36)<br><br>**Objective measures**<br>1) Daily physical activity (actigraphy)<br>2) Muscular strength (maximum isometric strength)<br><br>**Biological endpoints**<br>1) Serum creatine	**PROMs**<br>1) N.S.<br>2) N.S.<br><br>**Objective measures**<br>1) *p*<0.05<br>2) *p*<0.05<br><br>**Biological endpoints**<br>1) *p*<0.01	[91]
HRG80™<br>(n/a)	OPT<br>(188)	1994 CDC	Severe illness	Capsules of 200 mg HRG80 or tablets of 100 mg HRG80<br>One or two tablets/day<br>1 month	**PROMs**<br>1) Composite score of energy, wellbeing and mental clarity (VAS)*<br>2) Energy (VAS)<br>3) Sleep (VAS)<br>4) Pain (VAS)<br>5) Wellbeing (VAS)<br>6) Mental clarity (VAS)<br>7) Stamina (VAS)	**PROMs**<br>1) *p*<0.001<br>2) *p*<0.001<br>3) *p*<0.001<br>4) *p*<0.001<br>5) *p*<0.001<br>6) *p*<0.001<br>7) *p*<0.001	[110]
KPAX002<br>(n/a)	POC<br>(15)	1994 CDC	-	Methylphenidate: 5 mg BID the first 5 days, then scaled to 10 mg BID<br>Mitochondria formula: 4 tablets BID<br>3 months	**PROMs**<br>1) Fatigue (CIS total)*<br>2) Concentration disturbance (CIS subscale)<br>3) Fatigue (VAS)<br>4) Concentration (VAS)	**PROMs**<br>1) *p*<0.0001<br>2) *p*<0.0001<br>3) *p*<0.0001<br>4) *p*<0.0001	[119]

KPAX002<br>(NCT01966276)	RCT (PA)<br>(KPAX002: 63, Placebo: 65)	1994 CDC	-	Methylphenidate: 5 mg TID the first 5 days, then scaled to 10 mg TID<br>Mitochondria formula: 4 tablets TID<br>3 months	**PROMs**<br>1) Fatigue (CIS total)*<br>2) Fatigue (VAS)<br>3) Concentration disturbance (VAS)	**PROMs**<br>1) N.S.<br>2) N.S.<br>3) N.S.	[120]
L-carnitine and Amantadine<br>(n/a)	RCT (CO)<br>(28)	1994 CDC<br>Australian<br>Oxford	-	Two months of treatment with 1g TID L-carnitine or 100mg/day amantadine‡, 2 week washout period, two months in the opposite arm	**PROMs**<br>1) Fatigue (FSS)<br>2) Depression (BDI)<br>3) Psychiatric symptoms (SCL-90-R)<br>4) CFS impairment (CFS-II)<br>5) CFS severity (CFS-SI)	**PROMs**§<br>1) N.S. (4 weeks, 8 weeks)<br>2) $p<0.05$ (4 weeks, 8 weeks)<br>3) $p<0.05$ (4 weeks, 8 weeks)<br>4) $p<0.05$ (4 weeks, 8 weeks)<br>5) N.S. (4 weeks), $p<0.05$ (8 weeks)	[96]
NADH<br>(n/a)	RCT (CO)<br>(26)	1994 CDC	-	NADH 10 mg/day<br>1 month	**PROMs**<br>1) Symptom severity¶<br>**Biological endpoints**<br>1) Urinalysis (5-HIAA)	**PROMs**<br>1) $p<0.05$<br>**Biological endpoints**<br>1) $p<0.05$	[100]
NADH<br>(n/a)	RCT (PA)<br>(NADH: 36, Placebo: 41)	1994 CDC	-	NADH 20 mg/day<br>2 months	**PROMs**<br>1) Fatigue (VAS, FIS)<br>2) Functional impairment (KPS)<br>3) Mood (HAD)<br>4) Health related quality of life (SF-36)<br>5) Sleep (PSQI)<br>**Objective measures**<br>1) Heart rate<br>2) SBP<br>3) DBP	**PROMs**<br>1) N.S.<br>2) N.S.<br>3) $p<0.05$<br>4) N.S.<br>5) N.S.<br>**Objective measures**<br>1) $p<0.05$<br>2) N.S.<br>3) N.S.	[99]

Oxaloacetate<br>(NCT04592354)	OPT<br>(76)	1994 CDC	-	500 mg BID (n=23),<br>1,000 mg BID (n=29),<br>1,000 mg TID (n=24)<br>6 weeks	**PROMs**<br>1) Fatigue (CFQ)	**PROMs**<br>1) *p*<0.005	[98]
Ubiquinol-10<br>(n/a)	OPT<br>(20)	1994 CDC	-	Ubiquinol-10: 150 mg/day<br>2 months	**PROMs**<br>1) Fatigue (CFQ)<br>2) Depression (CES-D)<br><br>**Objective measures**<br>1) Arithmetic task (Uchida-Kraepelin Psychodiagnostic Test)<br>2) Sleep-wake cycle (Life Scope)<br>3) ANS function (APG)<br><br>**Biological endpoints**<br>1) CoQ10 levels<br>2) Oxidation activity (d-ROMs test),<br>3) Antioxidant activity (BAP test)	**PROMs**<br>1) N.S.<br>2) *p*<0.01<br><br>**Objective measures**<br>1) *p*<0.01<br>2) *p*<0.01<br>3) N.S.<br><br>**Biological endpoints**<br>1) *p*<0.001<br>2) N.S.<br>3) N.S.	[104]
Ubiquinol-10<br>(n/a)	RCT (PA)<br>(Ubiquinol-10: 14, Placebo: 17)	1994 CDC	-	Ubiquinol-10: 150 mg/day<br>3 months	**PROMs**<br>1) Fatigue (CFQ)<br>2) Depression (CES-D)<br><br>**Objective measures**<br>1) Arithmetic task (Uchida-Kraepelin Psychodiagnostic Test)<br>2) Sleep-wake cycle (Life Scope)<br>3) Autonomic nervous function (APG)<br><br>**Biological endpoints**<br>1) CoQ10 levels<br>2) Oxidation activity (d-ROMs test),	**PROMs**<br>1) N.S.<br>2) N.S.<br><br>**Objective measures**<br>1) *p*<0.05<br>2) N.S.<br>3) N.S.<br><br>**Biological endpoints**<br>1) *p*<0.001<br>2) N.S.	[104]

			3) Antioxidant activity (BAP test)	3) N.S.

Abbreviations:
1994 CDC: 1994 Centers for Disease Control and Prevention criteria for CFS by Fukuda, 5-HIAA: 5-hydroxyindoleacetic acid, ACR: American College of Rheumatology, ALC: acetyl L-carnitine, ANS: autonomic nervous system, APG: acceleration plethysmography, BAP: biological antioxidant potential, BDI: Beck depression inventory, BID: twice daily, BP: blood pressure, CES-D: Center for Epidemiologic Studies depression scale, CFS: chronic fatigue syndrome, CFS-II: chronic fatigue syndrome-impairment index, CFS-SI: chronic fatigue syndrome-severity index, CGI: clinical global impression of change, CIS: checklist individual strength, CoQ10: coenzyme Q10, CO: cross-over, CRP: C-reactive protein, DBP: diastolic blood pressure, DVAS: discrete visual analogue scale, DXA: dual X-ray absorptiometry, d-ROMs: reactive oxygen metabolite-derived compounds, FGF-21: fibroblast growth factor 21, FIS: fatigue impact scale, FMS: fibromyalgia syndrome, FSS: fatigue severity scale, GAA: guanidinoacetic acid, HADs: hospital anxiety and depression scale, HR: heart rate, HRG80™: hydroponically grown red ginseng, IL: interleukin, KPS: Karnofsky performance scale, n/a: not available, NAD+: oxidized form nicotinamide adenine dinucleotide, NADH: reduced form nicotinamide adenine dinucleotide, N.S.: not significant, NT-proBNP: amino-terminal pro-B type natriuretic peptide, MFI-20: 20-item multidimensional fatigue inventory, MPQ: McGill pain questionnaire, MPQ-DLV: McGill pain questionnaire-Dutch language version, OPT: open-label pilot study, PA: parallel arm, PLC: propionyl-L-carnitine, POC: proof of concept, PROMs: patient-reported outcome measures, PSQI: Pittsburgh sleep quality index, RCT: randomized placebo-controlled double-blinded clinical trial, SBP: systolic blood pressure, SCL-90-R: symptom checklist 90-R, Se: selenium, SF-12: 12-item short form survey, SF-36: 36-item short form health survey, SPPB: short physical performance battery, TID: three times a day, TNF-α: tumor necrosis factor-alpha, VAS: visual analogue scale, VCO_2: pulmonary carbon dioxide output, VO_2: pulmonary oxygen update.
Trial codes:
NCT: ClinicalTrials.gov
Footnotes:
* Marks primary outcome measure
† *p* values reported grouped FMS only, CFS only and FMS + CFS patients together
‡ 13 of 28 enrolled patients stopped taking amantadine due to side effects
§ *p* values only given for L-carnitine treatment
¶ Assessment using unpublished questionnaires created by the authors

Supplementary Table S4: Clinical trials investigating treatments for gastrointestinal disturbances in ME/CFS.

**Intervention (Trial code)**	**Study design (Cohort size)**	**Diagnostic criteria**	**Patient subgroup**	**Treatment regime**	**Outcome measures**	**Outcome significance**	**Reference**
Antibiotic (ACTRN12615000457549)	OPT (22)	CCC	Stool *Streptococcus* count > 10^5 CFU/g	400 mg Erythromycin, BID for 6 days	**PROMs**<br>1) Sleep (Day and sleep diary)<br>2) Mood (POMS-SF)<br>3) Symptom severity (SSH)<br><br>**Objective measures**<br>1) Sleep (actigraphy)<br><br>**Biological endpoints**<br>1) Stool *Streptococcus* count, *Enterococcus* count, *Lactobacillus* count, *Enterococcus %, Lactobacillus* %, *Bifidobacterium %*<br>2) Stool *Bifidobacterium* count<br>3) Stool *Streptococcus* %<br>4) Stool *Bifidobacterium %*	**PROMs**<br>1) *p*=0.020<br>2) N.S.<br>3) N.S<br><br>**Objective measures**<br>1) N.S.<br><br>**Biological endpoints**<br>1) N.S.<br>2) *p*=0.006<br>3) *p*=0.030<br>4) *p*=0.004	[143]
FMT (NCT04158427)	RCT (PA) (FMT: 5, Placebo: 6)	IoM	-	Bowel preparation using polyethylene glycol, followed by FMT of 30 g of fecal transplant from a universal donor (treatment) or the same patient (placebo) *via* colonoscopy	**PROMs**<br>1) Fatigue (VAS, MFIS)<br>2) Health related quality of life (15D, EQ-5D-3L)	**PROMs**<br>1) N.S.<br>2) N.S.	[160]
Leaky gut diet (n/a)	OPT (41)	1994 CDC	Increased IgM and/or IgA in response to LPS as confirmed in previous study (Maes *et al*. 2007)	Leaky gut diet, supplemented with L-carnitine, CoQ10, taurine and lipoic acid in case of carnitine and/or CoQ10 shortage, or curcumine and quercetine in case of systemic or intracellular inflammation.<br>Details of leaky gut diet and dosage of supplements not stated.	**PROMs**<br>1) Symptom severity (FFS)<br><br>**Biological endpoints**<br>1) Serum IgM against bacteria<br>2) Serum IgA against bacteria	**PROMs**<br>1) $p<0.0001$<br><br>**Biological endpoints**<br>1) $p \leq 0.020$†<br>2) N.S.	[136]
Probiotic (n/a)	RCT (PA) (Probiotic: 19, Placebo: 16)	CCC	Not bedridden	Probiotic (8 x 10^9 CFU of *Lactobacillus casei* Shirota), TID for 8 weeks	**PROMs**<br>1) Depression (BDI)<br>2) Anxiety (BAI)	**PROMs**<br>1) N.S.<br>2) $p<0.05$	[149]

Probiotic<br>(n/a)	OPT<br>(15)	1994 CDC	-	200 ml probiotic yogurt (10^8 CFU/ml of *Lactobacillus* F19, *Lactobacillus acidophilus* NCFB 1748, *Bifidobacterium lactis* Bb12), BID<br>30 days	**PROMs**<br>1) Symptoms (VAS, SF-12)	**PROMs**<br>1) N.S.	[151]
Probiotic<br>(n/a)	RCT (PA)<br>(Probiotic:28, Placebo: 20)‡	1994 CDC	No GI disorders	Probiotic (1 x 10^{10} CFU of *Bifidobacterium infantis* 35264), once per day for 8 weeks	**Biological endpoints**<br>1) Inflammation (plasma IL-6)<br>2) Inflammation (plasma TNF-α, CRP)	**Biological endpoints**<br>1) N.S.<br>2) *p*<0.05	[150]
Probiotic<br>(n/a)	OPT<br>(9)	1994 CDC	-	Week 1: Two cups of Enterelle (*Enterococcus faecium* UBEF-41, *Saccharomyces cerevisiae* spp. *boulardii, Lactobacillus acidophilus* LA 14)<br><br>Week 2: Two cups of Bifiselle (*Bifidobacterium longum, B. breve, B. bifidum, B. infantis*)<br>Rotanelle (*B. longum* AR81)<br><br>Week 3: Two cups of Ramnoselle (*L. rhamnosus GG*, *L. acidophilus*)<br>BID and two cups of Enterelle per day<br><br>Week 4 - 8: Two cups of Citogenex (*L. casei*, *B. lactis*) and two cups of Rotanelle per day	**PROMs**<br>1) Physical health (SF-36)<br>2) Mental health (SF-36)<br>3) Fatigue (CFQ)<br>4) Depression (BDI-I)<br>5) Depression (BDI-II)<br><br>**Biological endpoints**<br>1) Immunology (serum IgM)<br><br>2) Immunology (urinary free cortisol, plasma ROMs, serum IgG, serum IgA, ESR, DHEA-S, faecal calprotectin, serum CRP)	**PROMs**<br>1) N.S.<br>2) N.S.<br>3) N.S<br>4) N.S.<br>5) N.S.<br><br>**Biological endpoints**<br>1) *p*<0.05 (IgM increased)<br>2) N.S.	[153]
Probiotic<br>(ACTRN12614001077651)	OPT<br>(44)	CCC	Stool *Streptococcus* count > 3 x 10^5 CFU/g and > 5% of total anaerobic microorganisms	Week 1: baseline measurements<br><br>Week 2: 400 mg erythromycin BID<br><br>Week 3: 2 capsules of Pro4-50 (2.5 x 10^{10} CFU *Lactobacillus rhamnosus*, 1.5 x 10^{15} CFU *Bifidobacterium lactis,* 5 x 10^6 CFU *B. breve,* 5 x 10^6 CFU *B. longum*), per day<br><br>Week 4: 400 mg erythromycin BID	**PROMs**<br>1) Mood (POMS-SF)*<br>2) Mood (DASS-21)<br>3) Sleep efficiency (sleep diary)<br>4) Sleep (PSQI global score)<br>5) Sleep SOL, WASO (sleep diary)<br>6) Fatigue (MFI-20 subscale)<br>7) Brain fog (MTFQ subscale)<br>8) Total symptoms (SSH)<br><br>**Objective measures**	**PROMs**<br>1) N.S.<br>2) N.S.<br>3) *p*=0.035<br>4) *p*=0.027<br>5) N.S.<br><br>6) N.S.<br>7) N.S.<br>8) *p*=0.001<br><br>**Objective measures**	[152]

	Week 5: 2 capsules of Pro4-50, once per day	1) Sleep efficiency (actigraphy)*<br>2) Sustained visual attention (RVP-A')*<br>3) Sleep WASO (actigraphy)<br>4) Sleep SOL, restlessness/sleep fragmentation index (actigraphy)<br>5) Processing speed<br>6) Story memory<br>7) Cognitive flexibility<br>8) Verbal fluency<br>9) Word memory, spatial working memory, visual learning, planning)<br><br>**Biological endpoints**<br>1) Stool *Streptococcus* count<br>2) Stool *Bifidobacteria* and *Lactobacillus* count<br>3) D-lactate (urinary D-lactate:L-lactate ratio)	1) N.S.<br>2) *p*<0.001<br>3) *p*=0.007<br>4) N.S.<br>5) *p*=0.004<br>6) *p*=0.002<br>7) *p*=0.001<br>8) *p*=0.014<br>9) N.S.<br><br>**Biological endpoints**<br>1) *p*=0.003<br>2) N.S.<br>3) N.S.

Abbreviations:
15D: 15-dimension instrument for measuring health related quality of life, 1994 CDC: 1994 Centers for Disease Control and Prevention criteria for CFS by Fukuda, BAI: Beck anxiety inventory, BDI: Beck depression inventory, BDI-I: BDI version 1, BDI-II: BDI version 2, BID: twice daily, CCC: Canadian consensus criteria for ME/CFS by Carruthers, CFQ: Chalder fatigue questionnaire, CFU: colony-forming units, CRP: C-reactive protein, DASS-21: depression, anxiety and stress scale-21, DHEA-S: dehydroepiandrosterone sulfate, DSM: diagnostic and statistical manual of mental disorders, EQ-5D-3L: 3-level version of the EQ-5D developed by the EuroQol group, ESR: erythrocyte sedimentation rate; ESS: Epworth sleepiness scale; FFS: FibroFatigue scale, FMT: fecal microbe transplant, GI: gastrointestinal, HRQOL: health-related quality of life, IgA: immunoglobulin A, IgM, immunoglobulin M, IL-6: Interleukin-6; IoM: Institute of Medicine criteria, LPS: lipopolysaccharide, MAPI: multivariable apnoea prediction index; MFI-20: 20-item multidimensional fatigue inventory, MFIS: modified fatigue impact scale, MTFQ: multiple fatigue types questionnaire, n/a: not available, NCT: ClinicalTrials.gov study identifier, N.S.: not significant, OPT: open-label pilot study, PA: parallel-arm, POMS-SF: profile of mood states - short form, PROMS: patient-reported outcome measures, PSQI: Pittsburgh sleep quality index, RCT: randomized placebo-controlled double-blinded clinical trial, ROMs: reactive oxygen metabolite; RVP-A': rapid visual processing-A', SF-12: 12-item short form survey, SF-36: 36-item short form health survey, SOL: sleep onset latency, SSH: symptom severity and severity hierarchy chart, TID, three times a day, TNF-α: tumor necrosis factor-alpha, TST: total sleep time; VAS: visual analogue scale, WASO: wake after sleep onset.

Trial codes:
ACTRN: Australian and New Zealand Clinical Trial Registry, NCT: ClinicalTrials.gov

Foot notes:
* Marks a primary outcome measure
† Antibodies against *Hafnia alvei*, *Pseudomonas aeruginosa*, *Morganella morganii*, *Pseudomonas putida*, *Citrobacter koseri* and *Klebsiella pneumoniae*, were measured ($p = 0.005$, $p = 0.01$, $p = 0.004$, $p = 0.001$, $p = 0.001$ and $p = 0.02$, respectively)
‡ Apart from a ME/CFS cohort, study included cohorts of ulcerative colitis patients, psoriasis patients, and healthy controls

Supplementary Table S5: Clinical trials investigating treatments for neurological disturbances in ME/CFS.

**Intervention (Trial code)**	**Study design (Cohort size)**	**Diagnostic criteria**	**Patient subgroup**	**Treatment regime**	**Outcome measures**	**Outcome significance**	**Reference**
Duloxetine (NCT00375973)	RCT (PA) (Duloxetine: 30, Placebo: 30)	1994 CDC	Severity (MFI-20 general fatigue subscale of ≥13)	Duloxetine 60-120mg/day or placebo for 12 weeks	**PROMs**<br>1) General fatigue*, physical fatigue, reduced activity, reduced motivation (MFI-20 subscales)<br>2) mental fatigue (MFI-20 subscale)<br>3) Pain severity (BPI)<br>4) Pain interference (BPI)<br>5) Anxiety and depression (HADS)<br>6) Symptoms (CDC-SI)<br>7) Health related quality of life (SF-36)<br>8) Improvement (PGI-I)<br>9) Severity (CGI subscale)	**PROMs**<br>1) N.S.<br>2) p=0.01<br>3) p=0.05<br>4) p=0.03<br>5) N.S.<br>6) N.S.<br>7) N.S.<br>8) N.S.<br>9) p=0.02	[180]
Fluoxetine (n/a)	RCT (PA) (Fluoxetine, depressed: 21, Fluoxetine, non-depressed: 24, Placebo, non-depressed: 28, Placebo depressed: 23)	Oxford	Depressed patients (BDI score of ≥16)<br><br>Non-depressed patients (BDI score of ≤10)	20 mg/day Fluoxetine or placebo, for 8 weeks	**PROMs**<br>1) Subjective fatigue (CIS subscale and daily observed fatigue score)†*<br>2) Depression (BDI and SCL-90)†*<br>3) Symptoms (SIP)*<br>4) Physical Activity (CIS subscale and daily observed activity score)†*<br>5) Sleep (CIS subscale and daily observed sleep score)†*<br>6) Cognitive function (CIS subscale and SIP, daily observed memory and concentration score)†<br>7) Social interactions (SIP subscale)*<br>8) Self efficacy expectations (MHLC subscale and self observation list)†*	**PROMs**<br>1) N.S.<br>2) N.S.<br>3) N.S.<br>4) N.S.<br>5) N.S.<br>6) N.S.<br>7) N.S.<br>8) n/a	[177]

					**Objective measures**<br>1) Physical activity (Actometer) †*<br>2) Cognitive function (complex reaction-time test)*	**Objective measures**<br>1) N.S.<br>2) N.S.	
Fluoxetine and GET (n/a)	RCT (PA) (Fluoxetine and GET: 33, Placebo drug and GET: 34, Fluoxetine and placebo GET: 35, Placebo drug and placebo GET: 34)	Oxford	-	20 mg/day Fluoxetine or placebo, over six months.<br>Treatment GET involved physiotherapist appointments at weeks 0, 1, 2, 4, 8, 12, 20 and 26 where they received a prescribed exercise program with a physiotherapist receiving instructions to undertake their preferred aerobic activity for 20 minutes ≥3 times a week, with activity intensity increasing when there was a consistent HR reduction of 10 bpm post exercise and reduced perceived exertion.<br>Placebo GET involved participants attending physiotherapist appointments during which they discussed 7-day activity diaries but were not offered specific exercise guidance.	**PROMs**<br>1) Fatigue (CFQ)*<br>2) Health related quality of life (SF-36)<br>3) Anxiety and depression (HADS)<br><br>**Objective measures**<br>1) Muscle strength (HGS)<br>2) Functional work capacity‡ (ergometer)	**PROMs§**<br>1) N.S.<br>2) N.S.<br>3) *p*=0.04 (week 12), N.S. (week 26)<br><br>**Objective measures§**<br>1) n/a<br>2) N.S.	[178]
Fludrocortisone (n/a)	RCT (PA) (Fludrocortisone: 38, Placebo: 46)	1994 CDC	Neurally mediated hypotension (tilt table test)<br>Mild/moderate (≥65 on GWS)	Fludrocortisone acetate, titrated to 0.1 mg/day or placebo for 9 weeks	**PROMs**<br>1) Wellness (GWS)<br>2) Health related quality of life (SF-36)<br>3) Depression (BDI)<br>4) Mental Fatigue (WMFI)<br>5) Mood (POMS-SF)<br>6) Activity (DASI)<br><br>**Objective measures**<br>1) Tilt table testing	**PROMs**<br>1) N.S.<br>2) N.S.<br>3) N.S.<br>4) N.S.<br>5) N.S.<br>6) N.S.<br><br>**Objective measures**<br>1) N.S.	[202]
Fludrocortisone (n/a)	RCT (CO) (20)	1994 CDC<br>1988 CDC	-	6 weeks treatment with 0.1mg/day¶ fludrocortisone or placebo, 6 weeks washout period, 6 weeks in the opposite arm.	**PROMs**<br>1) Fatigue (VAS)*<br>2) Unrefreshing sleep (VAS)*<br>3) Muscle pains (VAS)*	**PROMs##**<br>1) N.S.<br>2) N.S.<br>3) N.S.	[203]

					4) Inability to concentrate (VAS)*<br>5) Headaches (VAS)*<br>6) Forgetfulness (VAS)*<br>7) Confusion (VAS)*<br>8) Joint pains (VAS)*<br>9) Painful lymph nodes (VAS)*<br>10) Sore throat (VAS)*<br>11) Light headedness (VAS)*<br>12) Depression (VAS)*<br>13) Distance before exhausted#*<br>14) Health related quality of life (SF-36)*<br>15) Mood status (PANAS)<br><br>**Objective measures**<br>1) Cognitive processing speed (Hicks paradigm reaction time test)<br>2) Treadmill (time to exhaustion)	4) N.S.<br>5) N.S.<br>6) N.S.<br>7) N.S.<br>8) N.S.<br>9) N.S.<br>10) N.S.<br>11) N.S.<br>12) N.S.<br>13) N.S.<br>14) N.S.<br>15) N.S.<br><br>**Objective measures##**<br>1) N.S.<br>2) N.S.	
Ginkgo and Cistanche (GkoCist) (NCT02807649)	RCT (PA) (Placebo: 64, Low dose: 62, High dose: 64)	1994 CDC	-	High dose (Ginkgo at 180mg/day and Cistanche at 450mg/day), low dose (Ginko at 120mg/day and Cistanche at 300mg/day), or placebo for 60 days.	**PROMs**<br>1) Physical fatigue (CFQ)<br>2) Mental fatigue (CFQ)<br>3) Physical health (WHOQOL subscale)<br>4) Psychological health (WHOQOL subscale)<br>5) Social relationships (WHOQOL subscale)<br>6) Living environment (WHOQOL subscale)<br>7) Sexual Life (SLQ)<br><br>**Biological endpoints**<br>1) Blood ammonia<br>2) Blood lactic acid<br>3) Liver and Kidney Function	**PROMS**<br>1) $p<0.0001$<br>2) $p<0.0001$<br>3) $p=0.0024$<br>4) N.S.<br>5) N.S.<br>6) N.S.<br>7) $p<0.0001$<br><br>**Biological endpoints**<br>1) $p=0.01$<br>2) $p=0.005$<br>3) N.S.	[195]

Low-dose phenelzine (n/a)	RCT (PA) (Phenelzine: 9, Placebo: 9)	1994 CDC	-	Phase 1: all participants took one placebo pill per day for 2 weeks.<br>Phase 2: active treatment group received alternating days of 15mg phenelzine pill and placebo pill for 2 weeks.<br>Phase 3: active treatment group received 15mg/day phenelzine for 2 weeks.<br>Placebo group took one placebo pill per day.	**PROMs**<br>1) Functional Status (FSQ)<br>2) Mood (POMS-SF)<br>3) Depression (CES-D)<br>4) Illness Severity (ISS)<br>5) Fatigue (FSS)<br>6) Symptoms (Sx)	**PROMS**<br>1) N.S.<br>2) N.S.<br>3) N.S.<br>4) N.S.<br>5) N.S.<br>6) N.S.	[175]
LDX (NCT01071044)	RCT (PA) (LDX: 15, Placebo: 11)	1994 CDC	Patients with executive impairment (measured using BRIEF-A)	30 mg/day LDX for 2 weeks, 50 mg/day LDX for 2 weeks, 70 mg/day LDX for 2 weeks (dose escalation in the absence of adverse events)	**PROMs**<br>1) Executive Function (BRIEF-A)<br>2) Fatigue (FSS)<br>3) Anxiety (HAM-A)<br>4) Pain (MPQ)<br>5) Fibromyalgia (FIQ)<br>6) ADHD (ADHDRS)<br>7) Severity (CGI subscale)	**PROMS**<br>1) p=0.005<br>2) p=0.008<br>3) N.S.<br>4) p = 0.046<br>5) N.S.<br>6) p=0.038<br>7) p = 0.022	[184]
MELATOZINC (NCT03000777)	RCT (PA) (Melatonin: 28, Placebo: 32)	1994 CDC	-	1mg Melatonin + 10 mg Zinc, 1 hour before sleep for 16 weeks	**PROMs**<br>1) Fatigue (FIS)<br>2) Anxiety and Depression (HADS)<br>3) Sleep Quality (PSQI)<br>4) Dysautonomia (COMPASS31)<br>5) Health related quality of life (SF-36)<br>**Biological endpoints**<br>1) 6-sulfatoxymelatonin<br>2) Zinc	**PROMS**<br>1) p<0.05<br>2) N.S.<br>3) N.S.<br>4) N.S.<br>5) N.S.<br>**Biological endpoints**<br>1) p<0.0001<br>2) N.S.	[191]
S-citalopram (n/a)	OPT (16)	1994 CDC	Co-morbid MDD	10-20 mg of S-citalopram daily for up to 12 weeks	**PROMs**<br>1) Fatigue (CFQ, FIS)<br>2) PEM (CFS-SR)<br>3) Sore throat (CFS-SR)<br>4) Tender lymph nodes (CFS-SR)<br>5) muscle pain (CFS-SR)<br>6) Joint pain (CFS-SR)<br>7) Headache (CFS-SR)<br>8) Sleep (CFS-SR)<br>9) Cognitive function (CFS-SR)<br>10) CFS severity (CGI subscale)	**PROMS**<br>1) p<0.0005<br>2) p=0.001<br>3) p=0.005<br>4) N.S.<br>5) p=0.001<br>6) p=0.025<br>7) p<0.0005<br>8) p<0.0005<br>9) p<0.0005<br>10) p<0.0005	[176]

11) CFS improvement (CGI subscale)	11) $p<0.0005$
12) Depression (HAM-D, BDI)	12) $p<0.0005$
13) MDD severity (CGI subscale)	13) $p=0.001$
14) MDD improvement (CGI subscale)	14) $p<0.0005$

Abbreviations:
1988 CDC: 1988 Centers for Disease Control and Prevention criteria for CFS by Holmes, 1994 CDC: 1994 CDC criteria for CFS by Fukuda, ADHDRS: attention deficit hyperactivity disorder rating scale, BAI: Beck anxiety inventory, BDI: Beck depression inventory, BP: blood pressure, BPI: brief pain inventory, BPM: beats per minute, BRIEF-A: behavior rating inventory of executive function-adult, CDC-SI: CDC symptom inventory scores, CES-D: Centers for Epidemiological Studies depression scale, CFQ: Chalder fatigue questionnaire, CFS-SR: CFS symptom rating, CGI: clinical global impressions, CO: cross-over, COMPASS31: composite autonomic symptom score 31-items questionnaire, CIS: checklist of individual strength, DASI: Duke activity status index, FIS: fatigue impact scale, FIQ: fibromyalgia impact questionnaire, FSQ: functional status questionnaire, FSS: fatigue severity scale, GET: graded exercise therapy, GWS: global wellness scale, HADS: hospital anxiety and depression scale, HAM-A: Hamilton anxiety rating scale, HAM-D: Hamilton depression rating scale, HGS: hand grip strength, HR: heart rate, ISS: illness severity scale, LDX: Lisdexamfetamine Dimesylate, MDD: major depressive disorder, MFI-20: 20-item multidimensional fatigue inventory, MHLC: multidimension health locus of control, MPQ: McGill pain questionnaire, n/a: not available, N.S. not significant, OPT: open-label pilot study, PA: parallel-arm, PANAS: positive and negative effect scale, PGI-I: patient global impression of improvement, POMS-SF: profile of mood states - short form, PROMs: patient-reported outcome measures, PSQI: Pittsburgh sleep quality index, RCT: randomized placebo-controlled double-blinded clinical trial, SF-36: 36-item short form health survey, SIP: sickness impact profile, SLQ: sexual life quality questionnaire, Sx: 16-question Symptom Checklist, UGWS: unidimensional global wellness scale, VAS: visual analogue scale, WHOQOL: World Health Organization quality of life questionnaire, WMFI: Wood mental fatigue inventory.

Trial codes:
NCT: ClinicalTrials.gov

Footnotes:
* Marks a primary outcome measure
† Total combined score from more than one measure to assess a domain
‡ Function work capacity defined as the amount of oxygen consumed in the last minute of exercise per kg of body weight
§ *p* values for fluoxetine treatment reported
¶ The dose of fludrocortisone was double if participants reported no improvement at week 2.
# Distance walked before exhaustion was ascertained using a 5-point rating scale: 1 point – 1 block, 2 points – 1-3 blocks, 3 points – 3 to 8 blocks, 4 points – 1 to 3 miles, 5 points – 3 miles or more.
Comparisons are between the active vs placebo phases of the study

Supplementary Table S6: Clinical trials investigating treatments for neuroendocrine disturbances in ME/CFS.

**Intervention (Trial code)**	**Study design (Cohort size)**	**Diagnostic criteria**	**Patient subgroup**	**Treatment regime**	**Outcome measures**	**Outcome significance**	**Reference**
CT38<br>(NCT03613129)	OPT<br>(17)	1994 CDC,<br>CCC and IoM	-	Subcutaneous infusion of CT38, with doses at:<br>0.20 µg/kg/hour, for 3 hours for 2 days<br>0.03 µg/kg/hour, for 3.5 hours for 3 days<br>0.06 µg/kg/hour, for 3.5 hours for 3 days<br>0.01 µg/kg/hour, for 3.5 hours for 3 days	**PROMs**<br>1) Symptoms (TDSS)<br>2) Physical Health (SF-36-PCS)<br>3) Mental Health (SF-36-MCS)	**PROMs**<br>1) $p=0.011$<br>2) $p=0.005$<br>3) N.S.	[235]
DHEA<br>(n/a)	OPT<br>(23)	1988 CDC	Suboptimal serum levels of DHEA-sulphate (<2.0µg/mL)	25 mg/day DHEA for 6 months†	**PROMs**<br>1) Functional disability (MHAQII)<br>2) Pain‡<br>3) Fatigue‡<br>4) Satisfaction with activity level‡<br>5) Coping with the illness‡<br>6) Feelings of helplessness‡<br>7) Depression‡<br>8) Anxiety‡<br>9) Thinking‡<br>10) Memory‡<br>11) Sexual problems‡<br><br>**Biological endpoints**<br>1) Free testosterone<br>2) Total cholesterol<br>3) LDL<br>4) HDL	**PROMs**<br>1) n/a<br>2) $p=0.035$<br>3) $p=0.009$<br>4) $p=0.033$<br>5) N.S.<br>6) $p=0.015$<br>7) N.S.<br>8) $p<0.01$<br>9) $p<0.01$<br>10) $p<0.05$<br>11) N.S.<br><br>**Biological endpoints**<br>1) $p=0.001$<br>2) N.S.<br>3) N.S.<br>4) $p=0.016$	[228]
GH<br>(n/a)	RCT (PA)<br>(GH: 10,<br>Placebo: 10)	1994 CDC	Nocturnal peak of <10 µg/L of GH	6.7 µg/kg GH per day for 12 weeks.<br><br>Following treatment period, the 17 patients remaining were given the above concentration of GH for 9 months	**PROMs**<br>1) Quality of Life (NHP, QoL-AGHDA)<br><br>**Objective measures**<br>1) Weight<br>2) Muscle Strength<br>3) Skinfold Thickness<br>4) Fat-free mass<br>5) Total body water<br>6) Fat mass<br>7) BMI	**PROMs**<br>1) N.S.<br><br>**Objective measures**<br>1) N.S.<br>2) N.S.<br>3) N.S.<br>4) $p=0.006$<br>5) $p=0.003$<br>6) N.S.<br>7) N.S.	[229]

					**Biological endpoints**<br>1) Hormone levels (IGF-I)<br>2) Hormone levels (Thyrotrophin, tri-iodo thyronine, thyroxine, prolactin, cortisol, follicle-stimulating hormone, lutenizing hormone, testosterone)<br>3) Serum Lipoprotein(a)<br>4) Amino acids (Tyrosine, valine, tryptophane, phenylalanine, isoleucine and leucine)	**Biological endpoints**<br>1) $p<0.001$<br>2) N.S.<br>3) $p=0.003$<br>4) $p<0.005$	
Low-dose hydrocortisone (n/a)	RCT (PA) (Hydrocortisone: 35, Placebo: 35)	1994 CDC	-	16 mg/m^2/day hydrocortisone per day, 20-30mg at 8AM and 5 mg at 2PM for 12 weeks	**PROMs**<br>1) Wellness (SI-GHS)<br>2) Mood (POMS)<br>3) Symptoms (SCL-90R)<br>4) Sickness (SIP)<br>5) Depression (BDI)<br>6) Activity (AS-10)<br><br>**Biological endpoints**<br>1) Resting and cosyntropin-stimulated cortisol levels	**PROMs**<br>1) $p<0.01$<br>2) N.S.<br>3) N.S.<br>4) N.S.<br>5) N.S.<br>6) N.S.<br><br>**Biological endpoints**<br>1) $p<0.01$	[225]
Low-dose hydrocortisone (n/a)	RCT (CO) (Hydrocortisone: 16, Placebo: 16)	1994 CDC		5 – 10 mg hydrocortisone, once per day for 28 weeks	**PROMs**<br>1) Fatigue (FS-11)<br>2) Disability (WSAS)<br>3) Health related quality of life (SF-36)<br>4) Psychological symptoms (GHQ)<br><br>**Biological endpoints**<br>1) 24h urinary free cortisol<br>2) Insulin stress test	**PROMs**<br>1) $p=0.04$<br>2) $p=0.006$<br>3) N.S.<br>4) $p=0.003$<br><br>**Biological endpoints**<br>1) N.S.<br>2) $p=0.52$	[226]

Abbreviations:
1988 CDC: 1988 Centers for Disease Control and Prevention criteria for CFS by Holmes, 1994 CDC: 1994 CDC criteria for CFS by Fukuda, ACTH: adrenocorticotropic hormone, AS-10: 10-point activity scale, BDI: Beck depression inventory, BMI: body mass index, CCC: Canadian consensus criteria for ME/CFS by Carruthers, CGI-21: clinical global impression scale-21, CO: cross-over, DHEA: dehydroepiandrosterone, FS-11: 11-item fatigue scale, GH: growth hormone, GHQ: general health questionnaire, hCRH: human corticotropin-related hormone, HDL: high density lipoprotein, IGF-I: insulin-like growth factor-I, IoM: Institute of Medicine, LDL: low density lipoprotein, MHAQII: modified health assessment questionnaire version 2, NCT: ClinicalTrials.gov study identifier, NHP: Nottingham health profile, n/a: not available, N.S.: not significant, OPT: open-label pilot study, POMS: profile of mood states, PROMS: patient-reported outcome measures, QoL-AGHDA: quality of life assessment in GH-deficient adults, RCT: randomized placebo-controlled double-blinded clinical trial, SCL-90R: Symptom Checklist-90-R, SF-36: 36-item short form health survey, SF-36-MCS: SF-36 – mental component summary, SF-36-PCS: SF-36 – physical component summary, SI-GHS: single item global health scale, SIP: sickness impact profile, TDSS: total daily symptom scores, WSAS: work and social adjustment scale.

Trial codes:
NCT: ClinicalTrials.gov
Footnotes:
† Dosage of DHEA increased by increments of 25mg/day to a maximum of 100mg/day in participants whose DHEA-sulphate levels remained <2 µg/mL or no clinical response
‡ Assessment tool not reported
